# Procedural recommendations of cardiac PET/CT imaging: standardization in inflammatory-, infective-, infiltrative-, and innervation (4Is)-related cardiovascular diseases: a joint collaboration of the EACVI and the EANM

**DOI:** 10.1007/s00259-020-05066-5

**Published:** 2020-10-27

**Authors:** Riemer H. J. A. Slart, Andor W. J. M. Glaudemans, Olivier Gheysens, Mark Lubberink, Tanja Kero, Marc R. Dweck, Gilbert Habib, Oliver Gaemperli, Antti Saraste, Alessia Gimelli, Panagiotis Georgoulias, Hein J. Verberne, Jan Bucerius, Christoph Rischpler, Fabien Hyafil, Paola A. Erba, 4Is Cardiovascular Imaging: a joint initiative of the European Association of Cardiovascular Imaging (EACVI), the European Association of Nuclear Medicine (EANM)

**Affiliations:** 1 Medical Imaging Centre, Department of Nuclear Medicine & Molecular Imaging, University of Groningen, University Medical Center Groningen, Groningen, The Netherlands; 2 Medical Imaging Centre, Department of Nuclear medicine & Molecular Imaging (EB50), University of Groningen, University Medical Center Groningen, Hanzeplein 1, 9700 RB Groningen, The Netherlands; 3 Faculty of Science and Technology Biomedical, Photonic Imaging, University of Twente, Enschede, The Netherlands; 4 Department of Nuclear Medicine, Cliniques Universitaires Saint-Luc, Brussels, Belgium; 5 Department of Surgical Sciences/Radiology, Uppsala University, Uppsala, Sweden; 6 Medical Imaging Centre, Uppsala University Hospital, Uppsala, Sweden; 7 British Heart Foundation Centre for Cardiovascular Science, University of Edinburgh, Edinburgh, UK; 8 Cardiology Department, APHM, La Timone Hospital, Marseille, France; 9 Aix Marseille Université, IRD, APHM, MEPHI, IHU-Méditerranée Infection, Marseille, France; 10 HeartClinic, Hirslanden Hospital Zurich, Hirslanden, Switzerland; 11 Turku PET Centre, Turku University Hospital, University of Turku, Turku, Finland; 12 Heart Center, Turku University Hospital, Turku, Finland; 13 Fondazione Toscana G. Monasterio, Pisa, Italy; 14 Department of Nuclear Medicine, Faculty of Medicine, University of Thessaly, University Hospital of Larissa, Larissa, Greece; 15 Department of Radiology and Nuclear Medicine, Amsterdam UMC, location AMC, University of Amsterdam, Amsterdam, The Netherlands; 16 Department of Nuclear Medicine, Georg-August University Göttingen, Göttingen, Germany; 17 Department of Nuclear Medicine, University Hospital Essen, University of Duisburg-Essen, Essen, Germany; 18 Department of Nuclear Medicine, DMU IMAGINA, Georges-Pompidou European Hospital, Assistance Publique - Hôpitaux de Paris, University of Paris, F75015 Paris, France; 19 PARCC, INSERM, University of Paris, F-75006 Paris, France; 20 Department of Nuclear Medicine, University of Pisa, Pisa, Italy; 21 Department of Translational Research and New Technology in Medicine, University of Pisa, Pisa, Italy

**Keywords:** PET/CT, 4Is, Cardiovascular diseases, Procedural recommendations

## Abstract

With this document, we provide a standard for PET/(diagnostic) CT imaging procedures in cardiovascular diseases that are inflammatory, infective, infiltrative, or associated with dysfunctional innervation (4Is). This standard should be applied in clinical practice and integrated in clinical (multicenter) trials for optimal procedural standardization. A major focus is put on procedures using [^18^F]FDG, but 4Is PET radiopharmaceuticals beyond [^18^F]FDG are also described in this document. Whilst these novel tracers are currently mainly applied in early clinical trials, some multicenter trials are underway and we foresee in the near future their use in clinical care and inclusion in the clinical guidelines. Finally, PET/MR applications in 4Is cardiovascular diseases are also briefly described. Diagnosis and management of 4Is-related cardiovascular diseases are generally complex and often require a multidisciplinary approach by a team of experts. The new standards described herein should be applied when using PET/CT and PET/MR, within a multimodality imaging framework both in clinical practice and in clinical trials for 4Is cardiovascular indications.

## Preamble

The European Association of Nuclear Medicine (EANM) is a professional nonprofit medical association that facilitates communication worldwide among individuals pursuing clinical and research excellence in nuclear medicine. The EANM was founded in 1985. The European Association of Cardiovascular Imaging (EACVI) promotes excellence in clinical diagnosis, research, technical development, and education in cardiovascular imaging to improve the standardization of CVI practice in Europe. The recently established joint cardiovascular imaging group (4Is joint collaboration group) between the EANM and the EACVI focuses on infiltrative, inflammatory, infectious, and innervation dysfunctional (4Is) cardiovascular diseases. This 4Is joint collaboration group is working on recommendations for imaging procedures in the field of 4Is cardiovascular diseases. These recommendations are intended to assist practitioners in providing appropriate (hybrid) nuclear medicine imaging. However, these are not inflexible rules or requirements of practice and are not intended, nor should these be used, to establish a legal standard of care. The ultimate judgment regarding the propriety of any specific procedure or course of action must be made by medical professionals taking into account the unique circumstances of each case. Thus, there is no implication that an approach differing from the recommendation, standing alone, is below the standard of care. To the contrary, a conscientious practitioner may responsibly adopt a course of action different from that set out in the recommendations when, in the reasonable judgment of the practitioner, such course of action is indicated by the condition of the patient, limitations of available resources or advances in knowledge or technology subsequent to publication of the recommendations. The practice of medicine involves not only the science but also the art of dealing with prevention, diagnosis, alleviation, and treatment of disease. The variety and complexity of human conditions make it impossible to always reach the most appropriate diagnosis or to predict with certainty a particular response to treatment. Therefore, it should be recognized that adherence to these recommendations will not ensure an accurate diagnosis or a successful outcome. All that should be expected is that the practitioner will follow a reasonable course of action based on current knowledge, available resources, and the needs of the patient to deliver effective and safe medical care. The sole purpose of these recommendations is to assist practitioners in achieving this objective.

## Introduction

Nuclear imaging plays a pivotal role in cardiac infectious, inflammatory, infiltrative, and innervation disorders. Cardiac amyloidosis, sarcoidosis, large vessel vasculitis (LVV), infective endocarditis (IE), infected cardiac implantable electronic devices (CIED), vascular graft infection (VGI), and myocardial innervation dysfunction are the main indications for the use of nuclear medicine procedures in both diagnosis and response assessment.

PET/CT and PET/MR imaging are noninvasive diagnostic tools that allow detection of radiopharmaceutical accumulation in tissues with high sensitivity and provide precise quantification of their local concentration. The most commonly used tracer at present is the fluorine-18–labeled glucose analogue [^18^F]-2-fluoro-2-deoxyglucose ([^18^F]FDG). [^18^F]FDG accumulation in tissues is proportional to their glucose utilization and reflects the glucose metabolism of cells. This glucose metabolism is increased in cancer but also in infectious and inflammatory processes (1). Anatomical and morphological information derived from the combination with CT (PET/CT) can be used to improve the localization, extent, and characterization of lesions detected by [^18^F]FDG PET. Beyond [^18^F]FDG, several other PET radiopharmaceuticals are available for imaging cardiovascular diseases. A major potential player in this field is [^18^F]-sodium fluoride ([^18^F]NaF) and [^68^Ga]DOTA conjugated peptides to somatostatin receptors (SSRs), showing promises for the evaluation of patients with atherosclerosis and heart valve disease (2, 3). The major advantage of [^18^F]NaF PET over [^18^F]FDG is the absence of any physiological myocardial uptake, thus making assessment of coronary arteries and valves in addition to the peripheral vasculature feasible.

Specific PET amyloid–binding radiotracers, structurally similar to thioflavin-T and likely binding to the amyloid fibril structure, are already approved for imaging beta amyloid in Alzheimer’s disease (4), such as [^11^C]-Pittsburgh compound B ([^11^C]PiB), [^18^F]florbetapir and [^18^F]florbetaben. They have been recently successfully used to image cardiac amyloidosis (5, 6).

[^11^C]-Hydroxyephedrine ([^11^C]mHED) is the most widely used PET tracer for cardiac presynaptic sympathetic imaging. In a healthy heart, there is a homogeneous distribution of [^11^C]mHED over the left ventricle, making it a valuable tracer for detecting specific regional defects of the presynaptic sympathetic system in disease (7) such as heart failure and arrhythmias.

Currently, the use of PET/CT in cardiovascular diseases is mainly derived and adapted from published oncological imaging procedures and guidelines for which the European Association Research Ltd. (EARL) has established a standard (8, 9) for intercenter harmonization for [^18^F]FDG imaging. A standard for PET/CT imaging in inflammatory, infective, infiltrative, and innervation dysfunctional (4Is) cardiovascular diseases is currently lacking. Therefore, standards for PET imaging not limited to FDG are needed specifically designed for cardiovascular disorders.

### Goals

The purpose of this document is to assist in performing PET/CT and PET/MR for cardiovascular imaging in the field of 4Is, starting from the selection of the proper radiopharmaceutical based on the specific patients’ clinical condition and extending to the correct use of imaging acquisition protocols, postprocessing, interpretation, and reporting. As PET is a quantitative imaging technique, specific quality control (QC)/quality assurance (QA) procedures are required to maintain the accuracy and precision of quantitation (10), and these aspects are also included. Indeed, repeatability and reproducibility are essential requirements for any quantitative measurement and in establishing the clinical value of imaging biomarkers. For quantification of [^18^F]FDG PET/CT and PET/MR standardized uptake values (SUVs) are the most commonly used semiquantitative parameters for tracer uptake analysis. Proposing a standardized imaging procedure will promote the appropriate use of PET/CT and PET/MR imaging in clinical practice, increase the quality of investigator driven clinical trials and allow comparison between studies thereby contributing to evidence-based medicine. This document is built upon earlier published European procedural guidelines for quantitative [^18^F]FDG PET and PET/CT in oncology (8) and infectious and inflammatory diseases (1, 11, 12).

### Clinical indications in cardiovascular diseases

[^18^F]FDG PET/CT and PET/MR have an increasingly relevant role in inflammation and infection imaging; they are rapidly evolving imaging modalities (1). However, no appropriateness criteria have been developed to date for these indications in cardiovascular diseases. It must be emphasized that the level of evidence available at this time for using PET/CT and PET/MR imaging with either [^18^F]FDG or novel PET radiopharmaceuticals varies for many of the indications described in this document, but randomized controlled trial data (as with most forms of cardiovascular imaging) are consistently lacking.

General indications for 4Is cardiovascular PET/CT include:

- Noninvasive diagnosis
- Imaging-guided biopsy diagnosis
- Therapy response
- Monitoring
- Prognosis

Specific routine clinical practice and clinical research applications:

- Prosthetic and native valve infective endocarditis (IE) (clinical) (11, 13)
- Cardiac implantable electronic (CIED) and left ventricular assist devices (LVAD) (clinical) (11, 13)
- Vascular graft infection (VGI) (clinical) (14)
- Cardiac sarcoidosis (clinical) (15)
- Large vessel vasculitis (LVV) (clinical) (16, 17)
- Cardiac amyloidosis (clinical research) (5, 6)
- Atherosclerosis and valvular disease (clinical research) (18)
- Myocardial innervation (clinical research) (19)

Note: Clinical means PET imaging is used in routine clinical practice based upon clinical evidence and guideline recommendations; the clinical research phase refers to PET imaging techniques that do not currently meet the above criteria but are undergoing clinical research evaluation in patients. However, even the above techniques used in the routine clinical practice require further research for optimization and ideally randomized controlled trials to establish their clinical utility.

### Radiopharmaceuticals (Table 1)

Most PET radiopharmaceuticals are labeled with ^18^F, but some are labeled with the shorter living ^11^C (T_1/2_ 20 min), or are generator produced ^68^Ga (T_1/2_ 68 min). The most promising radiopharmaceutical developments include the application of existing tracers such as [^18^F]NaF in atherosclerosis, and the use of radiolabeled compounds for detection of cardiac amyloidosis ([^18^F]florbetaben, [^18^F]florbetapir, [^18^F]flutemetamol, and [^11^C]PiB). [^68^Ga]DOTA conjugated peptide ([^68^Ga]DOTATOC, DOTATATE, DOTANOC) compounds with affinity to SSRs, and [^18^F]FLT, hold promise in detecting cardiac sarcoidosis with the advantage of having no physiological myocardial uptake which is the main disadvantage of [^18^F]FDG. The [^18^F]-labeled sympathetic nerve PET radiopharmaceuticals [^18^F]LMI1195 (generic name [^18^F]flubrobenguane) are promising with potential to aid clinical decision making, e.g., for optimal selection of patients requiring an ICD or CRT. An overview of PET radiopharmaceuticals for 4Is beyond [^18^F]FDG is given in Table 1.

**Table 1:** PET cardiovascular tracers (4Is)

PET tracer	Cardiovascular indication	Clinical	Dose activity	Effective dose^ (uSv/MBq) (112)	Imaging p.i.	Imaging mode	Imaging duration	Reconstruction method	Reconstructed pixel size^#^/matrix size	Remarks
[^18^F]-FDG (11, 15, 18, 34, 113–126)	Vasculitis, endocarditis/ CIED, vascular graft, sarcoidosis, AL amyloidosis, atherosclerosis	Clinical Not clinical*	2.5–5.0 MBq/Kg 3.0–7.0 MBq/Kg**	19	45–120 min	2D / 3D	2–4 min per bed*** 5–10 min per bed (3D data collection)** 20–30 min per bed (2D data collection)**	Iterative 2D/3D reconstruction algorithm, OSEM post-filtering Time-of-flight Point spread function correction	Between 1.0 to 5.3 × 1.0 to 5.3 × 1.0 to 3.4 mm^3^	* AL amyloidosis, atherosclerosis. ** Cardiac sarcoidosis. *** Depending on vendor suggestion of camera system.
[^18^F]-NaF (41, 127–132)	Amyloidosis, atherosclerosis Valve disease	Clinical trial	125–370 MBq 1.5–3.7 MBq/Kg	24	60–180 min	3D	2–5 min per bed	OSEM post-filtering	Between 1.0 to 5.3 × 1.0 to 5.3 × 1.0 to 3.4 mm3 matrix size 128 × 128	For coronary imaging suggest performing scans with CT coronary angiogram
[^18^F]-fluorocholine (133)	Atherosclerosis	Clinical trial	4 MB/kg	28	30–60 min	3D, dynamic, static	1 bed position	Iterative reconstruction	Matrix size 128 × 128	
[^18^F]-PEG-folate (134)	Atherosclerosis	Clinical trial	185 MBq	17	1–60 min	2D / 3D, dynamic, static	3 min per bed	Iterative reconstruction	Matrix size 128 × 128	
[^18^F]-FLT (135, 136)	Sarcoidosis	Clinical	3.7–5 MBq/Kg	15	45–60 min	3D	10 min	OSEM post-filtering Time-of-flight Point spread function correction	Between 1.0 to 5.3 × 1.0 to 5.3 × 1.0 to 3.4 mm3	
[^18^F]-florbetapir	Amyloidosis	Clinical trial	169–370 MBq	19	60–90 min	3D, Static, List mode	3 min per bed	Iterative reconstruction Post-filtering Time-of-flight Point spread function correction	Between 1.0 to 5.3 × 1.0 to 5.3 × 1.0 to 3.4 mm3	
[^18^F]-florbetaben (83, 138, 139)	Amyloidosis	Clinical trial	222–375 MBq 4 MBq/Kg	19	60–145 min	3D, static, list mode	20–120 min 2 min per bed	OSEM post-filtering Time-of-flight	Between 1.0 to 5.3 × 1.0 to 5.3 × 1.0 to 3.4 mm3	Dynamic imaging and whole-body imaging
[^18^F]flutemetamol (38)	Amyloidosis	Clinical trial	360 MBq	35	0–30 min	3D, static, list mode	1 bed position	Iterative reconstruction	Matrix size 200 × 200	Static images of the last 20 min dynamic imaging
[^18^F]-flubrobenguane (140, 141)****	Regional innervation	Clinical trial	3 MBq/kg	26	5–60 min	2D or 3D Static	5–10 min	Iterative reconstruction or FBP	3–4 mm	****Also known as LMI-1195
[^68^Ga]-SST (3, 142, 143)	Sarcoidosis, atherosclerosis	Clinical trial	150-250 MBq 2–4 MBq/Kg	21–25	60–90 min	2D, 3D, static	4 min per bed Cardiac region: 20 min (additional)	Iterative reconstruction	2.74 × 2.74 × 3.27 mm	Probably of lower value in cardiac sarcoidosis
[^11^C]mHED (40)	Regional innervation	Clinical trial	200–400 MBq	5.6	Dynamic, with injection	2D, 3D, list mode	60 min	Iterative reconstruction	2.2 × 2.2 mm2	
[^11^C]PiB (21, 36, 37, 49)	Amyloidosis	Clinical trial	5 MBq/kg	4.7	Dynamic, with injection	3D list mode	30 min	Iterative reconstruction	2.34 × 2.34 × 3.27 mm	PIB SUV and RI correlate well with BP_ND_ which is assumed to be proportional to amyloid concentration

^**#**^Values for reconstructed pixel size of [^18^F]-radiotracers are based on the literature for [^18^F]FDG

^^^Without low dose CT

*p.i.* post-injection, *CIED* cardiac implantable electronic device, *AL amyloidosis* immunoglobulin light chain amyloidosis, *OSEM* ordered subset expectation maximization, *RI* retention index, *SST* somatostatin, *mHED* metahydroxyephedrine, *PiB* Pittsburgh compound-B, *SUV* standardized value, *BP*_*ND*_.binding potential

### Administered activity (Table 1)

#### [^18^F]FDG PET

The administered activity is not crucial for the results of the exam but should be within a certain range depending also on the type of PET scanner. The EANM guidelines on [^18^F]FDG PET imaging in inflammation/infection suggest an administered activity of 2.5–5.0 MBq/kg, which is concordant with 175–350 MBq or 4.7–9.5 mCi for a 70-kg standard adult. In the USA, the recommended [^18^F]FDG administered activity is 370–740 MBq (10–20 mCi) for adults and 3.7–5.2 MBq/kg (0.10–0.14 mCi/kg) for children (1).

#### [^18^F]NaF

In adults, the recommended [^18^F]NaF injected activity for bone imaging is 1.5–3.7 MBq /kg with a maximum recommended activity of 370 MBq for obese patients. Most cardiovascular imaging studies have been conducted with either 125 or 250 MBq.

#### [^18^F]florbetaben, [^18^F]florbetapir, [^18^F]flutemetamol, [^11^C]PiB

[^18^F]florbetaben (300 MBq), [^18^F]florbetapir (370 MBq), [^18^F]flutemetamol (180–360 MBq), and [^11^C]PiB (5 MBq/kg) might be injected as a single intravenous slow bolus in a total volume of 10 mL (20, 21).

#### [^68^Ga]DOTA-conjugated peptides to SSRs (DOTATOC, DOTATATE, DOTANOC)

The administered activity ranges from 150 to 250 MBq, also depending on the characteristics of the PET camera system. The recommended activity to obtain good quality images is at least 150 MBq (3).

#### [^11^C]mHED

A recommended activity of 370 MBq is injected as a slow bolus.

## PET/CT procedure

### Qualifications and responsibilities of personnel

In Europe, the certified nuclear medicine physician who performs the study and authorizes the report is responsible for the whole procedure, according to national laws and rules. Patient preparation and imaging procedures should be executed by qualified nuclear medicine technologists (http://www.eanm.org/content-eanm/uploads/2016/11/EANM_2017_TC_Benchmark.pdf).

### General patient preparation

Whole body PET, see also Boellaard et al. (8), EANM Nuclear Medicine Guide (www.EANM.org/Publications).

### [^18^F]FDG

#### [^18^F]FDG suppression in normal myocardium

To suppress physiological [^18^F]FDG uptake in the normal myocardium, it is recommended to use patient preparation protocols including high-fat–enriched diet lacking carbohydrates for 12–24 h prior to the scan combined with a prolonged fasting period of 12–18 h, with or without the use of intravenous heparin of 50 IU/kg approximately 15 min prior to [^18^F]FDG injection (11, 22, 23). In addition, strenuous exercise should be avoided for at least 12 h prior to the exam. Following [^18^F]FDG injection, and before the images are obtained, the patient should continue to fast and should restrain from any physical activity, as both will enhance myocardial glucose uptake.

#### Blood glucose level

It has been advocated that high serum glucose levels may interfere with the detection of inflammatory and infectious sites due to competitive inhibition between [^18^F]FDG uptake and circulating D-glucose. However, a study by Rabkin et al. (24) demonstrated that neither diabetes nor hyperglycemia at the time of the study had a significant effect on the false-negative rate in infection and inflammation imaging. Therefore, although all efforts should be made to decrease blood glucose to a normal level, hyperglycemia (< 11 mmol/L, or < 180 mg/dl) in patients with unstable or poorly controlled diabetes, this should not represent an absolute contraindication for performing the study if clinically indicated (11). [^18^F]FDG should be injected no sooner than 4 h after subcutaneous injection of rapid-acting insulin or 6 h after subcutaneous injection of short-acting insulin. [^18^F]FDG administration is not recommended on the same day after injection of intermediate-acting and/or long-acting insulin (8).

#### Concomitant treatments

Although antimicrobial treatment for cardiac infection is expected to decrease the intensity of inflammation and therefore [^18^F]FDG accumulation (25), there is currently no evidence to routinely recommend treatment discontinuation before performing PET/CT. The risk of false negative [^18^F]FDG PET scans is probably lowest, if the patient is imaged when their CRP is > 40 mg/L (26).

In contrast, inflammatory disorders treated with steroids can lead to false-negative results (27). Delaying the commencement of steroid treatment till after the scan is therefore strongly recommended, whenever clinically safe, with exception for giant cell arteritis due to risk of vision loss. If this is not feasible or if the patient is already on steroids, then one should aim at performing the exam as soon as possible after steroid initiation (preferentially within 3 days) or at the lowest possible dose that is safe. Similar principles apply to other immunosuppressive treatments. Monitoring therapy effect under steroids and/or immunosuppressive drugs may reduce the FDG signal on PET.

#### Other special considerations

[^18^F]FDG imaging can be performed in patients with kidney failure, although image quality may be suboptimal and prone to interpretation pitfalls (28). Creatinine and/or glomerular filtration should be evaluated, according to national guidelines, if intravenous contrast agent will be administered. If renal function is impaired and [^18^F]FDG PET/CT examination with intravenous CT contrast agent is deemed necessary, then adequate prevention of nephrotoxicity should be performed according to local or society guidelines (see paragraph, Contrast-enhanced CT procedurals in 4Is).

#### Specific patient preparation non[^18^F]FDG radiopharmaceuticals

In general, there are no dietary restrictions, and fasting is not needed. Patients should be well hydrated to promote rapid excretion of the radiopharmaceutical in order to reduce radiation dose and to improve image quality.

#### [^18^F]florbetaben, [^18^F]florbetapir, [^18^F]flutemetamol, [^11^C]PiB

There is no known evidence suggesting any drug interactions between amyloid radiotracers and common drugs used in patients with amyloidosis. Therefore, no specific dietary preparation (no restriction on oral intake) or withholding of medication is recommended at this time.

#### [^68^Ga]DOTA-conjugated peptides to SSRs

Some authors suggested withdrawal of cold SST analogues (whenever possible and not contra-indicated) to avoid potential SST receptor blockade which will improve uptake. In this case, the time interval between interruption of therapy and [^68^Ga]DOTA-conjugated peptide PET/ CT depends on the type of drugs used: 1 day is suggested for short-lived molecules and at least 3–4 weeks for long-acting analogues (29).

#### [^11^C]mHED

The following medication should be discontinued in any case: reserpine, cocaine, tricyclic antidepressants, calcium channel blockers, labetalol, and tranquilizers (especially phenothiazines). In the literature, interferences with other drugs have been reported and should be taken into account, comparable to [^123^I]mIBG (30).

#### [^18^F]-NaF

No specific preparation needed***.***

### PET acquisition (Table 1)

#### [^18^F]FDG

In general, the same acquisition, reconstruction and postprocessing steps as those described in the EANM procedure guideline for tumor imaging with [^18^F]FDG PET/CT (8) are recommended for 4Is cardiovascular [^18^F]FDG PET/CT imaging.

Shortly, these are, as stated earlier, the recommended [^18^F]FDG administered activity is between 175 and 350 MBq. In general, 2-min/bed position for an injected activity of 3 MBq.kg^−1^ are used. For modern, high sensitivity scanners with axial field-of-view (FOV) of 20 cm or more, time-of-flight (TOF), and improved image reconstruction methods, the administered activity (or scan time per bed position) can be reduced by a factor 2 compared to the aforementioned numbers. For patients weighing more than 90 kg, it is recommended to increase the scan time per bed position, instead of further increasing the administered activity.

The time interval between [^18^F]FDG injection and scanning is critical if semiquantification using SUV is intended, but less important for visual reading only. Although the recommended interval is 60–90 min for cardiovascular imaging (similar to tumor imaging), 120–180 min is sometimes applied to help assess inflammatory activity in the vascular wall and left ventricle due to lower background activity in the blood pool (18, 31, 32), but these extended time-intervals seem less effective in infection detection (33). The regional acquisition time can be doubled for optimal visualization of small vascular structures, as with cranial and neck arteries in vasculitis (34).

Especially for attenuation correction of the thorax, a respiration-averaged low-dose CT can be considered, as this will likely give better alignment between PET and CT over the heart. Other than that, the recommendations for low-dose CT attenuation correction for tumor imaging with [^18^F]FDG can be followed.

Adding gated cardiac PET for 4Is indications is optional. It may improve image quality, particular in coronary atherosclerosis assessment and (prosthetic) valve infective IE, but supporting literature for [^18^F]FDG is scarce (35).

Whole-body [^18^F]FDG PET imaging is particularly useful in patients with a suspicion of whole body involvement of the primary disease and to identify septic embolism, mycotic aneurysms, and the portal of entry.

#### Specific PET acquisition (Table 1)

For cardiac sarcoidosis, it is highly recommended to complement inflammation imaging with perfusion imaging (generally nuclear imaging looking for perfusion defects) or scar imaging (cardiac magnetic resonance) in order to assess the presence of both active inflammation and scar (15).

For amyloidosis and innervation imaging with specific tracers such as [^18^F]florbetaben, [^18^F]florbetapir, [^18^F]flutemetamol, [^11^C]PiB, and [^11^C]mHED respectively, a dynamic scan starting simultaneously with tracer injection covering the heart has to be performed covering the heart starting simultaneously with radiopharmaceutical injection, followed by a static image reconstruction (Table 1): [^11^C]PIB: 10–20 min p.i. (36, 37); [^18^F]florbetapir: 10–30 min p.i. (20); [^18^F]flutemetamol: 0–30 min p.i. (38); [^18^F]florbetaben: 0–20 min p.i. (5); [^11^C]mHED: 0–60 min p.i. (39, 40). Acquisition should be in list mode, or, if list mode is not available, should be done as a dynamic scan with framing that allows for calculation of the retention index (see later) at the desired interval. [^11^C]mHED can be combined with rest perfusion imaging for evaluating match/mismatch patterns. For amyloidosis, the dynamic scan can be followed by a whole body scan in order to assess extracardiac uptake.

[^18^F]NaF imaging in atherosclerosis is recommended 60 min after [^18^F]NaF administration (41, 42). Delayed imaging, 3 h after tracer administration, may improve signal to noise but involves more complex imaging logistics (43). When imaging the coronary arteries, ECG gating should be performed with motion correction of the [^18^F]NaF PET data to reduce image noise, and increase the PET signal (43) This should be coupled with contrast-enhanced CT coronary angiography to facilitate accurate coregistration of the PET and CT data sets in three dimensions. For superior coronary motion control and radiation exposure reduction, a prospective diastolic acquisition should be used together with the administration of beta blockers for heart rate control. This approach allows assessment of adverse plaques and disease activity in the coronary arteries with [^18^F]NaF PET (43). Similar protocols are recommended for [^18^F]NaF imaging of carotid atheroma, abdominal aortic aneurysms, and both native and bioprosthetic valve diseases (44–46).

For [^68^Ga]DOTA-conjugated peptides in the setting of atherosclerosis, acquisition 60 min after administration results in optimal vascular wall activity against blood pool background (3). Similar protocols to those suggested for [^18^F]NaF, including motion correction and contrast CT coronary angiography (CTA), should be applied for coronary imaging.

### PET image reconstruction (Table 1)

#### [^18^F]FDG

In general, images should be reconstructed according to the guidelines for tumor imaging with [^18^F]FDG PET/CT (8), using iterative reconstruction with a product of subsets and iterations between 40 and 60. Use of TOF and resolution recovery is recommended as it has been shown to improve disease detectability in cardiac PET (47).

All corrections necessary to obtain quantitative images should be applied during the reconstruction. More advanced image reconstruction methods, such as penalized reconstruction, are possible; however, the use of these methods is rather limited to visual assessment and should not be used interchangeably with regular iterative reconstruction methods (48).

#### Specific PET image reconstruction (Table 1)

[^18^F]NaF reconstruction, included gating, is comparable to [^18^F]FDG PET/CT imaging, although motion correction and careful coregistration with CTA is recommended, particularly for coronary and valve imaging. For [^68^Ga]DOTA-conjugated peptides, standard reconstructions are adequate, although coronary imaging requires motion correction and coregistration with CT coronary angiography as described for [^18^F]NaF. Amyloid ligands static images: same reconstruction settings as for [^18^F]FDG. For amyloid ligands, the dynamic scans need to be reconstructed into frames of increasing duration to allow for calculation of the retention index. For [^11^C]PIB, the scan can be rebinned into two static frames (0–15 and 10–20 min).

### PET data analysis

#### [^18^F]FDG

Image quality for myocardial evaluation: overall quality (good, average, low), motion artifacts, abnormal biodistribution, quality of [^18^F]FDG suppression in the myocardium (full suppression, partial suppression, unsuppressed). Standard commercial software programs can be applied for reading and quantifying [^18^F]FDG data. Cardiovascular images can be displayed in the standard three views (short, 2× long views/axes), or polar maps can be generated for example for amyloid and innervation imaging. Quantification of PET data on different software programs should be done with caution, due to variability in results.

For assessment of uptake in the large vessels, e.g., in vasculitis and atherosclerosis, the [^18^F]FDG uptake pattern (diffuse or focal uptake), the exact location of the uptake, the extent of the uptake/vascular segments, and the intensity (vascular scoring 0–3 against the liver) should be scored.

We also recommend quantification of [^18^F]FDG activity, using SUVmax, SUVpeak, SUVmean, and application of target-to-background (TBR) analysis (with vascular blood pool as reference) (18).

#### Specific PET data analysis

For cardiac amyloidosis, regions of interest (ROI) need to be drawn over the left ventricular wall and in the center of the left atrium (circular ROI with a diameter of four pixels defined in the left atrium near the valve plane on at least three neighboring slices) to derive myocardial uptake and the arterial input curve, respectively (36). Further segmentation into myocardial regions (anterior, inferior, septal and lateral) can be done as preferred. For analysis of amyloidosis and innervation imaging, specific software programs are needed, such as PMOD, Matlab, Carimas, and aQuant. For calculation of the retention index (RI), the myocardial tracer uptake at a certain time after injection has to be divided by the area under the curve of the blood pool activity up to that point in time. This provides a quantitative measure of amyloid binding or innervation depending on the tracer injected. For example, the RI for [^11^C]PIB is defined as the ratio between the tissue activity concentration at ~ 10–20 min and the integral of the blood pool curve between 0 and 15 min; whilst the RI for [^18^F]florbetapir is the ratio of activity concentration between 10 and 30 min and the integral of the blood pool curve between approximately 0 and 20 min. Alternatively, calculation of SUV ratio between myocardial wall and blood pool can be done for the 10–20 min interval for [^11^C]PIB or 10–30 min interval for [^18^F]florbetapir. A SUV cutoff of 1.09 or RI cutoff of 0.037 min^−1^ is used to discriminate patients with cardiac amyloidosis from healthy subjects when using [^11^C]PIB (37), compared to 1.45 and 0.025 min^−1^ for [^18^F]florbetapir (20). These cutoff values are valid only for the given time intervals and tracers. It should be noted that amyloidosis patients and healthy controls could also be discriminated completely by visual assessment using the 10–20 min [^11^C]PIB images (37, 49). From the arterial and myocardial time-activity curves, retention indices and washout (WO) rates for [^11^C]mHED can be calculated. The cardiac RI reflects norepinephrine recycling from the synaptic cleft. RI values were determined for the global left ventricle (LV) and for the anterior, lateral, inferior, and septal wall segments. [^18^F]NaF analysis for atherosclerosis in the large vessels is comparable to [^18^F]FDG PET quantification. Quantification of [^18^F]NaF across the coronary vasculature currently relies on SUVmax and TBRmax measurements in individual plaques (with blood pool measured in the atria), although novel assessments quantifying tracer activity across the entire coronary vasculature are in development (50).

For [^68^Ga]DOTA-conjugated peptides, the analysis is comparable to [^18^F]FDG PET quantification in atherosclerosis. In short, within each 2D ROI, maximum voxel value [^68^Ga]DOTA activity will be derived to estimate maximum tissue-to-blood ratio (TBRmax), normalized by mean blood pool activity measured within five consecutive circular ROIs drawn within the lumen of the superior vena cava (3).

## Contrast-enhanced CT procedurals in 4Is (Table 2)

For a diagnostic CTA standard CT settings as suggested by related guidelines and the supervising radiologist or responsible physician should be employed (8). Medication potentially interacting with intravenous contrast agents (e.g., metformin) and relevant medical history (e.g., compromised renal function) should be taken into consideration. Since patients of the 4I’s can be considered at risk, renal function should generally be assessed in this group of patients before administration of contrast agents because of possible nephrotoxicity. Patients with a higher risk of contrast agent induced nephrotoxicity are patients with an eGFR <  30 ml/min/1.73 m^2^ (51). In these patients, the use of less nephrotoxic contrast agents or a renalguard system might be considered. In general, the laboratory values obtained should not be older than 6 months at the time of scan. Other risk factors for kidney damage caused by contrast agents include dehydration or volume depletion, the intake of nephrotoxic substances, an age above 70 years, and existing cardiovascular diseases. The discontinuation of NSAIDs and aminoglycosides also reduces the risk of impaired renal function by contrast administration. Furthermore, attention must be paid to patients with a history or possible history of previous contrast agent hypersensitivity reactions. Premedication with glucocorticoids and H1- and H2-blockers reduce the risk of an anaphylactic reaction, but unenhanced CT should generally be preferred in patients with a known severe contrast reaction. In general, PET/CT may be performed without the administration of contrast agent in patients with suspected cardiac amyloidosis or altered myocardial innervation as the accumulation of these tracers is highly specific to the respective imaging target. In cardiac sarcoidosis, different imaging protocols exist: if a perfusion examination is performed in addition to [^18^F]FDG PET, a contrast-enhanced CT is usually not necessary. However, CTA may be helpful to better assign [^18^F]FDG uptake to the myocardium or surrounding structures (lungs, lymph nodes, etc.).

**Table 2:** Interpretation of contrast**-**enhanced CT scans acquired alongside PET/CT imaging in 4Is

Disease	Contrast application	Advantages and scoring methods	Comments
Infective endocarditis and cardiac device infection	++	-Visualization of abscesses -Visualization of thrombi/vegetation on valves/probes -Visualization of septic embolism as infarcts in terminal vessels (e.g., spleen, kidney, brain) -Detailed examination of valves (potentially important in surgical procedures)	Some imaging centers do not deem the administration of contrast medium to be mandatory.
Cardiac sarcoidosis	±	Superior morphological allocation of the PET signal (e.g. myocardial vs. lung uptake; organ involvement)	Contrast agent generally not required if perfusion study (PET and SPECT) is available
Large vessel vasculitis	++	Visualization of the vessels to exclude relevant stenosis and score wall thickness: 0 = no mural thickening 1 = slight mural thickening 2 = mural thickening 3 = long and strong circumferential mural thickening OR as measurement: >2–3 mm	In the presence of a recent angiographic scan (CT/MRT), a low-dose CT is sufficient.
Atherosclerosis	+++	Visualization and quantification of calcium, vascular stenosis and plaque composition -Agatston score in mainly applied for calcium burden and risk assessment in coronary artery disease -Vascular stenosis is evaluated on CTA and categorized as non-obstructive or obstructive	CTA is clinically recommended and aids in the interpretation of the PET scans particularly in the coronary arteries
Vascular graft infection	+++	-Visualization of peri-graft gas and fluid. -Aneurysm expansion/pseudo-aneurysm formation -Detailed examination of vascular graft	The sensitivity and specificity of CT is moderate and variable
Cardiac amyloidosis	–	Assessment of thickness of the left ventricular myocardium	Only patients with a clinical suspicion receive this specific examination (septum thickness usually already available).

^-^no contribution, ^±^some contribution, ^++^good contribution, ^+++^excellent contribution

In case of infective endocarditis (IE) and cardiac implantable electronic devices (CIED) infection, combining [^18^F]FDG PET with/CT angiography (CTA) is helpful in the identification of a larger number of anatomic lesions and in reducing the number of equivocal scans (52, 53). Optional is the use of diluted contrast. The diluted contrast may help defining the 4 heart chambers better, and make anatomic localization of endocarditis easier (triphasic contrast administration for better delineation of the right and left cardiac chambers). In particular, CTA can help in the diagnosis of pseudoaneurysm, fistulas, and abscesses associated with infected valves and for the accurate assessment of valve prostheses. CTA is especially useful in patients with aortic grafts, or congenital heart diseases and complex anatomy. Another advantage is that in case of IE of the aortic valve, CTA can provide useful information about the anatomy of the valve, such as the size or extent of any calcification of the valve and ascending aorta, as it can also differentiate between pannus vs thrombus/vegetation in case of elevated transvalvular pressure gradients. This information is important for a proper surgical management. In addition, ceCT imaging might facilitate the diagnosis of septic embolisms in both left-sided infective endocarditis (abdomen and brain) and right-sided infective endocarditis (pulmonary). The technical requirements for performing PET/CTA with a hybrid PET/CT scanner are cardiac gating for both techniques and at least a 64-detector row CT. For the evaluation of left-sided prosthetic IE, an arterial phase ECG-gated CTA must be performed. When PET/CTA is performed to diagnose device infection, a prospective, ECG-gated, venous phase CTA sequence is recommended to evaluate local soft tissue changes, lead vegetation, and venous thrombosis of the vascular accesses. In case of vasculitis, CTA is helpful to evaluate the arterial wall thickness for primary diagnosis, and to monitor vascular stenosis during disease progression (16, 54) (Table 2). CTA is clinically applied for the evaluation of plaque composition and characterization of high risk plaques, the degree of luminal stenosis and vascular calcifications using nonenhanced CT in atherosclerotic disease, with coronary CTA widely used in the assessment of patients presenting with chest pain (55–57) (Table 2).

## [^18^F]FDG PET/CT data assessment, interpretation, and reporting 4Is: adapted from Jamar et al. (1)

### General assessment of [^18^F]FDG PET

At the end of the PET acquisition and before image interpretation, image quality should be verified.

The level of noise should be low. If the level of noise is too high, the physician should check if the total [^18^F]FDG activity injected to the patient was adapted to the body weight, verify that the residual activity at the level of the venous catheter is low, and confirm the absence of patient motion during the acquisition. If image quality is poor, PET acquisitions should be repeated, using a longer frame duration in cases of low [^18^F]FDG activity.

In the presence of high [^18^F]FDG uptake in peripheral muscles, patients should be asked about carbohydrate consumption and/or insulin injection in the 6-h preceding [^18^F]FDG injection. In the presence of high residual blood signal, blood glucose at the time of [^18^F]FDG injection should be checked. It is recommended to inject [^18^F]FDG when the blood glucose is < 11 mmol/L, or < 180 mg/dl, see section PET/CT procedure.

Suppression of [^18^F]FDG signal in the myocardium should be evaluated. Physiological myocardial [^18^F]FDG uptake usually occurs in a diffuse intense pattern across the myocardium but can also demonstrate regional variation. In absence of adequate myocardial suppression of the [^18^F]FDG signal, the compliance of the patient to the preparative procedures should be checked, and this information included in the report.

For the interpretation of PET acquisitions, it is important to make sure that the registration between PET and CT acquisitions is good, in particular, in the cardiac region. In presence of misregistration, data should be realigned, or ultimately, if realignment is not successful, an additional acquisition should be made for both PET and CT focused on the myocardium only. [^18^F]FDG PET findings should ideally be discussed by a multimodality team with expertise in both the diagnostics and management of patients with a suspicion of 4Is cardiovascular diseases.

### General visual analysis

Data can be evaluated with commercially available software systems. Both CT-attenuation corrected and non-corrected PET images have to be evaluated in the coronal, transaxial, and sagittal planes, as well as in tridimensional maximum intensity projection (MIP) cine mode. FDG-PET images are visually analyzed by assessing increased myocardial [^18^F]FDG uptake, taking into consideration the pattern (focal, focal on diffuse, linear, diffuse), intensity, and relationship to areas of physiologic distribution in the near surroundings. PET information should always be compared with morphologic information available on CT, including ceCT scans where available. It must be kept in mind that the sensitivity of [^18^F]FDG for infection and inflammation is not absolute and that even in the case of negative PET results, a thorough interpretation of the ceCT scan is essential.

### General quantitative analysis (SUV)

In contrast to its use in oncology, SUV has only been partly validated in inflammation and infection. Therefore, SUV metrics should be used with caution in clinical practice, particularly regarding the use of specific SUV cutoff values. In a [^18^F]FDG PET study in IE, a SUV cutoff > 3.3 was suggested to avoid false-positive findings (26). However, extrapolation of this cut-off value to other cardiovascular disease states is difficult, in part due to differences in the underlying pathophysiology and the intensity of inflammation. Moreover, care has to be taken when extrapolating absolute SUV cutoff values acquired between different hospitals and scanners because of the variation in these values related to differences in the scanner and reconstruction methods used.

### General interpretation criteria

To evaluate clinical [^18^F]FDG PET-CT imaging, the following should be taken into consideration:

- Clinical question
- Clinical history: fever, infection, inflammatory/auto-immune symptoms
- Prior imaging findings
- Brief treatment history, with particular regards to the presence of cardiac/vascular devices, date of implantation/extraction, surgical/postsurgical complications
- Concomitant treatment including date of initiation/withdrawal of antimicrobial therapy, steroids, statins, beta-blockers, etc.
- Biomarkers: CRP/ESR value at the time of imaging, results of blood cultures (number of positive blood culture, germ type)
- Scanning protocol (± cardiac gating, CTA)
- Adequate patient preparation.
- Physiologic distribution of [^18^F]FDG, and evaluation of its individual variations in the specific patient
- Localization of abnormal uptake according to anatomic imaging data.
- The presence and aspect of the [^18^F]FDG signal (focal / diffuse and homogeneous / heterogeneous) and persistence of PET signal on non-attenuation corrected (NAC) images. The presence of a focal, heterogenous [^18^F]FDG signal that persists on non-attenuation corrected PET images is an imaging aspect in favor of an infectious process.
- Intensity of [^18^F]FDG uptake (e.g., SUVmax)
- Correlation with data from previous clinical, biochemical, and morphologic examinations
- Presence of potential causes of false-negative results (lesion size, low metabolic rate, hyperglycemia, lesions masked by adjacent high physiologic uptake, concomitant drug use interfering with uptake, such as ongoing steroid therapy in systemic disorders)
- Presence of potential causes of false-positive results (injection artifacts and external contamination, reconstruction artifacts from attenuation correction, use of surgical glue in previous operations, normal physiologic uptake, pathologic uptake not related to infection or inflammation)

## Specific scoring, interpretation, and reporting criteria for 4IS disorders

### Prosthetic and native valve endocarditis and cardiac devices

IE comprises native valve endocarditis and infection of intracardiac prosthetic material. The latter includes prosthetic valve endocarditis (PVE, covering all types of prosthetic valves, clips, annuloplasty rings, intracardiac patches, and shunts), and infective endocarditis related to CIED, which include pacemakers, implantable cardioverter defibrillators (ICDs), and LVADs.

### Prosthetic valve endocarditis

#### Study indication

Suspected PVE, and/or septic embolisms, spread of infection, and portal of entry (POE).

#### Image analysis and interpretation

The location, pattern, and intensity of the [^18^F]FDG signal at the valve: intravalvular (in the leaflets), valvular (following the supporting structure of the valve) or perivalvular (next to the valve) (58). A perivalvular signal is in favor of infection, but infection cannot be excluded in the presence of intra-valvular or valvular [^18^F]FDG signal. Focal and heterogenous uptake is consistent with an infected valve. A typical location for abscesses in PVE is the aorto-mitral trigon, but abscesses can develop in any region in contact with prosthetic material. The probability of infection increases with the intensity of the [^18^F]FDG signal at the valves/prosthesis. The previous use of surgical adhesives can result in false positive scan findings soon after valve surgery. Post-operative inflammation can also lead to a false positive scan, but depending on the level of risk for infection (26) and a noncomplicated valve surgery, scans < 3 weeks surgery can be considered.

Several metrics have been tested to quantify the [^18^F]FDG signal in prosthetic valve endocarditis. The easiest semiquantitative parameter to measure is the highest SUV (SUVmax) in the valvular region. Another semiquantitative parameter that has been proposed is the prosthetic to background ratio (PBR) that takes into account the variability of the signal related to residual blood pool activity and image noise, by correcting valve SUV values by background activity in remote nonaffected myocardium.

Whole body [^18^F]FDG PET imaging is particularly useful in patients with a suspicion or proven PVE to identify septic embolism, mycotic aneurysms, and the POE.

[^18^F]FDG PET is less suited to detect cerebral septic embolism and mycotic aneurysms of intracerebral arteries owing to the high physiological uptake of [^18^F]FDG in the brain. In these cases, CT or MRI is the exam of choice.

Septic emboli appear as focal areas of [^18^F]FDG uptake and are typically located in the spleen, the liver, the lungs, and the kidneys. Uptake at the intervertebral disks and/or the vertebral bone (spondylodiscitis) suggests metastatic infection, which can also be observed in muscles and joints (septic arthritis). Embolic events can be clinically silent in 20% of cases, especially those affecting the spleen or brain. Septic emboli appear typically on CTA as hypodense lesions. [^18^F]FDG PET is more sensitive and specific than CTA for the detection of septic emboli (11, 59).

[^18^F]FDG PET imaging in IE is also useful to identify the POE. Typical portals of entry that can be identified are dental abscesses, sinusitis, infected central catheters, skin infection, and colonic cancers/polyps (11, 59).

In order to facilitate the interpretation of [^18^F]FDG PET images, we suggest classification of the [^18^F]FDG findings as follow (13, 60, 61):

Typical findings

- Presence of focal, heterogenous, valvular/peri-valvular [^18^F]FDG uptake persisting on NAC images and corresponding to an area of suspected infection on echocardiography or CTA (mobile mass, perivascular thickening, aneurysm, or new perivalvular regurgitation).
- High [^18^F]FDG signal in the absence of prior use of surgical adhesives.
- Presence of focal [^18^F]FDG uptake in organs with low-background uptake consistent with septic embolism, mycotic aneurysms or the portal of entry (POE)

Atypical findings

- Diffuse, homogeneous, valvular [^18^F]FDG signal that is absent on NAC images
- Low [^18^F]FDG signal

In all cases, correlation with clinical features echocardiography and CT findings is mandatory. In doubtful cases, white blood cell single-positron emission tomography (WBC-SPECT) can further help define the presence/absence of infection at PVE. In patients who present with suspected native valve endocarditis (NVE), the use of [^18^F]FDG-PET/CT is less well established. The relatively low sensitivities of FDG PET/CT reported in the literature for evaluation of NVE can be accounted for by both physiological and technical factors (63). The more frequent presence of isolated valve vegetation, rare para-valvular involvement, lower predominance of polymorphonuclear cells, and increased fibrosis in NVE compared with PVE results in reduced inflammatory response and subsequently lower FDG uptake (64). Notably, the lower sensitivity of FDG PET/CT is offset by a near perfect specificity for detection of NVE and its unrivaled ability to identify septic emboli (63). Thus, FDG PET/CT might provide clinically useful information and beneficially impact management in a subset of patients with suspicion of NVE, and the application of gated-PET may further improve it (35). The study indication, image analysis and interpretation are in general comparable with PVE.

### Infection of cardiac implantable electronic devices (CIED)

#### Study indication

- Suspected infection of CIED
- Defining the extent of infection in a proven CIED infection
- Positive blood culture in a patient with CIED

#### Image analysis and interpretation

Presence and aspect of the [^18^F]FDG signal (focal/linear) and persistency on NAC images. The presence of a focal or linear [^18^F]FDG signal that is located on or alongside a lead on CT and persists on NAC images are characteristics in favor of an infectious process. Late PET acquisitions might prove particularly useful in case of persistent high blood signal on PET images acquired at 1 h p.i. CIED infection might be confined to the leads, the pocket, or involve both sites. From a clinical perspective, it is important to differentiate superficial incisional infection which does not require CIED system extraction, from infection limited to the pocket, and those extending to the leads which are commonly associated with systemic infection and/or IE (65, 66). Lead extraction is a high risk procedure, associated with a risk of emboli, major bleeding including cardiac tamponade that increases with the time since device implantation, in addition damage to the tricuspid valve and resultant significant tricuspid valve regurgitation and deterioration of the right heart side function, these complications and can be avoided if the infection is limited to the pocket or incision. Therefore, in CIED infections the presence of [^18^F]FDG uptake should be described as pertinent to generator pocket (superficial or deep) and/or to the leads (intravascular or intracardiac portion of the leads). In addition, signs of cardiac (valvular or pericardial) involvement as well as systemic signs of infections (septic embolism, in particular, in the lung parenchyma and POE) should be carefully assessed and reported.

The presence of [^18^F]FDG uptake along pacing leads, in particular in the same location as mobile elements on echocardiography and in association with septic pulmonary emboli appearing as multiple focal [^18^F]FDG spots, is highly suggestive of pacing lead infection (67). The contrast between [^18^F]FDG signal along the pacing lead and residual blood signal is usually improved with delayed PET acquisitions (3 h p.i) (68). In addition, every positive blood culture should be carefully evaluated and prompt active exclusion of CIED infection with other diagnostic techniques (69).

The pattern and intensity of [^18^F]FDG uptake should be described considering that:

- Moderate [^18^F]FDG uptake in relation to post-operative residual inflammation can be found up to 2 months after CIED implantation but is usually of lower intensity than in case of infection.
- A focal [^18^F]FDG signal is often present at the point of entry of the lead into the subclavian vein that resembles an focal inflammation. The semiquantitative ratio of maximum activity concentration of the pocket device over mean count rate of lung parenchyma (67) or normalization of SUVmax around the CIEDs to the mean hepatic or blood pool activity (70) might help in differentiating mild postoperative residual inflammation up to 2 months after device implantation versus infection.
- The presence and location of the signal and its persistency on NAC PET images should be described according to the signal intensity and its location.

For CTA analysis and interpretation, see Table 2.

The evaluation of remote septic emboli should be performed similar to cases of prosthetic valve endocarditis, but with close attention also paid to the lung parenchyma.

In doubtful cases, white blood cell single-positron emission tomography (WBC-SPECT) can further help define the presence/absence of infection at PVE (11, 13, 69).

### Left ventricular assist device infection

#### Study indication

- Suspected infection of LVAD
- Evaluation of the extent of infection of LVAD
- Positive blood culture in a patient with LVAD

#### Image analysis and interpretation

LVADs are generally subdivided into 5 regions that have to be assessed separately: driveline exit site, driveline within the subcutaneous tissues, LVAD pump, LVAD inflow cannula, and LVAD outflow cannula.

- The presence, intensity and location of the [^18^F]FDG signal across the different components of the device and the persistency of the signal on NAC images should be described (71).
- The analysis of the FDG signal in the pump and cannula is more complex because of the artifacts caused by the device. The persistence of [^18^F]FDG uptake on NAC and its association with infiltration around the pump on the nonenhanced CT is highly suggestive of infection. In doubtful cases, WBC-SPECT can help define the presence/absence of infection of the pump and cannula (72).

Infection of the driveline can be treated by reimplantation of a new driveline in another site, whereas infection of the pump and cannula usually requires long-term antibiotic therapy.

### Vascular graft infection

VGI is a rare but severe complication after vascular surgery, associated with high morbidity and mortality rates (73). Early diagnosis of VGI is important for correct and early surgical and/or antibiotic treatment, which improves the outcome. Aortic grafts are frequently used at the time of valve surgery, with infection of valves and grafts often coexisting.

Recently, the European Society for Vascular Surgery (ESVS), in collaboration with the EANM, published clinical practice guidelines for the care of patients with vascular graft/endograft infection (14).

#### Study indication

- Diagnosis of suspected VGI

#### Image analysis and interpretation

The following aspects need to be carefully considered. Vascular graft uptake pattern, focal [^18^F]FDG uptake is more consistent with infection than diffuse low-level activity. The exact location of the focal uptake, its distribution and intensity should be recorded as well as [^18^F]FDG uptake in regional lymph nodes. The intensity of [^18^F]FDG accumulation can be assessed visually using a scoring system of 0–4: grade 0, [^18^F]FDG uptake similar to the background; grade I, low [^18^F]FDG uptake, comparable with that by inactive muscles and fat; grade II moderate [^18^F]FDG uptake, clearly visible and higher than the uptake by inactive muscles and fat; grade III, strong [^18^F]FDG uptake, but distinctly less than the physiologic urinary uptake by the bladder; and grade IV, very strong [^18^F]FDG uptake, comparable with the physiologic urinary uptake by the bladder. Focal uptake, with an intensity grade > II is suspected of vascular graft infection (74). However, in addition to visual assessment, [^18^F]FDG uptake should also be quantified with SUVmax for all arterial graft territories and normalized for background activity in the liver or blood pool usually in the caval vein. Diffuse, homogeneous, and low intensity [^18^F]FDG uptake can be observed in the majority of noninfected vascular graft prostheses particularly shortly after surgery. This is related to the body’s response to foreign material, and should be considered to avoid misinterpretation of PET/CT studies in patients referred for suspected prosthetic infection (75).

Whole body imaging describe remote locations in the body with abnormal increases in [^18^F]FDG uptake. Mycotic aneurysm appears typically as a focal [^18^F]FDG signal in a region corresponding to the arterial wall of the aorta or a peripheral artery and should be confirmed with CTA.

Comparison with prior [^18^F]FDG PET scans, if the scan is performed to determine response to therapy, then the distribution and intensity of the signal should be compared to prior scans: increase in uptake, no change in uptake, decrease in uptake.

Abnormalities on low dose CT should also be described. For CTA analysis and interpretation, see Tables 2 and 4. In doubtful cases, WBC-SPECT can further help define the presence/absence of infection at the vascular graft (76).

### Cardiac sarcoidosis

The role of [^18^F]FDG PET for the diagnosis of extracardiac sarcoidosis is well established. The assessment of cardiac sarcoidosis is more complex but is now recommended for clinical use by international guidelines (15, 77). Serial assessment of the inflammatory status using [^18^F]FDG PET might be helpful for monitoring therapy efficacy and for deciding treatment continuation, tapering, or change of treatment.

#### Study indication

- Suspicion of cardiac sarcoidosis according to the HRS guidelines (77)
- Monitoring of treatment in patients with established cardiac sarcoidosis

#### Image analysis and interpretation

Left ventricle: uptake pattern (1—no [^18^F]FDG uptake, 2—diffuse [^18^F]FDG uptake, 3—focal [^18^F]FDG uptake, 4—focal on diffuse [^18^F]FDG uptake; exact location of the focal uptake; extent of the uptake; intensity of the uptake).

Right ventricle: uptake pattern (grades 1–4), exact location of the focal uptake, extent of the uptake, intensity of the uptake.

Combination of [^18^F]FDG and perfusion imaging (MPI): Perfusion defects in patients with cardiac sarcoidosis can represent areas of scar or inflammation. However perfusion defect in combination with abnormal [^18^F]FDG uptake represents focal inflammation (Table 3) and can help differentiate pathological from physiological [^18^F]FDG activity. [^18^F]FDG and MPI patterns have been described as ‘early’ (only [^18^F]FDG-positive),‘progressive inflammatory’ ([^18^F]FDG positive without major perfusion defects); ‘peak active’ (high [^18^F]FDG uptake with small perfusion defects), ‘progressive myocardial impairment (high [^18^F]FDG uptake with large perfusion defects) or ‘fibrosis-predominant’ ([^18^F]FDG negative, but with perfusion defects) (15). In patients with areas of increased [^18^F]FDG uptake but no clear perfusion defects, this may represent either early cardiac sarcoid beyond the resolution of perfusion imaging, or false positive physiological [^18^F]FDG uptake.

**Table 3:** Interpretation of combined rest perfusion and [^18^F]FDG imaging in cardiac sarcoidosis (Adapted from Slart et al. (15))

Rest perfusion	[^18^F]FDG	Interpretation
Normal perfusion and metabolism		
Normal	No uptake	Negative for cardiac sarcoidosis
Normal	Diffuse	Diffuse (usually homogeneous) [^18^F]FDG most likely due to suboptimal patient preparation
Normal	Isolated lateral wall uptake	May be a normal variant
Abnormal perfusion or metabolism		
Normal	Focal	Could represent early disease or false positive
Defect	No uptake	Perfusion defect represents scar from sarcoidosis or other etiology
Abnormal perfusion and metabolism		
Defect	Focal in area of perfusion defect	Active inflammation with scar in the same location
Defect	Focal on diffuse with focal in area of perfusion defect	Active inflammation with scar in the same location with either diffuse inflammation or suboptimal preparation
Defect	Focal in area of normal perfusion	Presence of both inactive scar and inflammation in different segments of the myocardium or inactive scar and false positive physiological [^18^F]FDG uptake

As an alternative to MPI, [^18^F]FDG PET can be compared with CMR late gadolinium enhancement images. Areas of increased [18F]FDG that correspond to noninfarct areas of subepicardial and midmyocardial late gadolinium enhancement typically in the septum and lateral wall are highly suggestive of active cardiac sarcoidosis. Areas of typical late gadolinium enhancement with no [^18^F]FDG uptake are consistent with scarred, nonactive sarcoid regions. Regions of [^18^F]FDG uptake without late enhancement either representing early sarcoidosis beyond the sensitivity of CMR or false positive physiological [^18^F]FDG activity. Myocardium with neither increased [^18^F]FDG nor late enhancement is considered as normal (78).

Whole-body imaging describe extracardiac locations with increased [^18^F]FDG uptake. Comparison with prior [^18^F]FDG PET scan, if scan is performed in the context of assessing therapy response, then both the distribution and intensity should be compared to prior scans (increase, equal, or decreased uptake). SUV quantification can be applied in cardiac sarcoidosis diagnosis, which may provide prognostic information (79). Abnormalities on low dose or CTA scan should be described (Table 2). Comparison with other imaging modalities: cardiac MRI and echocardiography. CMR has limited value to assess treatment response because the majority of these patients receive intracardiac devices that may preclude CMR or produce artifacts when a MR compatible ICD is implanted.

[^68^Ga]DOTA conjugated peptides maybe promising as alternative cardiac sarcoidosis, with the benefit of no physiological myocardial uptake. [^68^Ga]DOTA conjugated peptides can either be scored visually for intensity and distribution, or semiquantitatively using SUVs (80).

### Large vessel vasculitis

LVV is defined as a disease mainly affecting the large arteries, with two major variants, Takayasu arteritis (TA) and giant cell arteritis (GCA). Vasculitis can be distributed locally in the branches of the external carotid artery or the aorta and its main branches more centrally in the thorax. Recent recommendations and statements have been provided, based on the available evidence and consensus of experts in the field, describing patient preparation, as well as [^18^F]FDG PET/CT(A) acquisition and interpretation for the diagnosis and follow-up of patients with suspected or diagnosed LVV (16, 17).

In circumstances where there may be cardiac involvement, patients with LVV should be further investigated (additional myocardial perfusion imaging, CMR, CT coronary angiography). This includes the risk of cardiovascular toxicity related to drug therapy used in LVV (81).

#### Study indication

- Diagnosis of LVV
- Monitoring of LVV activity

#### Image analysis and interpretation

Large vessels as well as the cranial and extracranial arterial structures. Uptake pattern diffuse circumferential [^18^F]FDG uptake around the vessel, that is different from the more regional and focal uptake observed in atheroma. The exact location of the uptake, its distribution across the vascular system, and its intensity (vascular scoring 0–3 against the liver) should be documented (Table 4). Whole-body imaging describe extravascular locations with increased [^18^F]FDG uptake. Comparison with prior [^18^F]FDG -PET scans, if the scan is performed to assess response to therapy, then extent and intensity should be compared to prior scans: increase in uptake, equal uptake, decrease in uptake. Abnormalities on low dose CT should be described. Comparison with other imaging modalities: CTA or MRA if available. For CTA analysis and interpretation, see Table 2.

**Table 4:** Recommended [^18^F]FDG PET/CTA interpretation criteria in LVV (Adapted from Slart et al. (16))

LVV visual grading (GCA and TA)		
[^18^F]FDG	Grade 0	No vascular uptake (≤ mediastinum)
	Grade 1	Vascular uptake > mediastinum and < liver uptake
	Grade 2	Vascular uptake = liver uptake, may be PET-positive
	Grade 3	Vascular uptake > liver uptake, considered PET-positive

*LVV* large vessel vasculitis, *GCA* giant cell arteritis, *TA* Takayasu arteritis

### Cardiac amyloidosis (clinical research)

Most cases of cardiac amyloidosis result from two protein precursors: amyloid immunoglobulin light chain (AL), in which the misfolded protein is a monoclonal immunoglobulin light chain typically produced by bone marrow plasma cells, and amyloid transthyretin (ATTR) amyloidosis, in which the misfolded protein is transthyretin (TTR), a serum transport protein for thyroid hormone and retinol that is synthesized primarily by the liver. [^18^F]FDG PET is mainly applied in AL cardiac amyloidosis, and may be supportive of the usual diagnostic tests in differentiating between systemic amyloidosis (no increased FDG uptake at the amyloid site) and localized amyloidosis (increased FDG uptake at the amyloid site) (82). However, other more specific PET radiopharmaceuticals such as [^11^C]PIB, [^18^F]florbetapir, and [^18^F]florbetaben, have demonstrated promise in clinical research studies as recently described in the Expert Consensus Recommendations for Multimodality Imaging in Cardiac Amyloidosis (5). In general, AL demonstrates a higher retention of these specific PET compounds as compared with ATTR cardiac amyloidosis (20, 83). SPECT imaging with bone tracers is generally preferred for the assessment of ATTR amyloidosis, although some preliminary research data have suggest [^18^F]NaF PET might provide similar results to SPECT bone agents but with the opportunity for tracer quantification (5, 84).

### Study indication

- Mainly research

#### Image analysis and interpretation

Left ventricle: uptake pattern (1—no [^18^F]FDG uptake (none), 2—diffuse [^18^F]FDG uptake (diffuse), 3—focal [^18^F]FDG uptake (focal), 4—Focal on diffuse [^18^F]FDG uptake (focal on diffuse), exact location of the focal uptake, extent of the uptake, intensity of the uptake.

Right ventricle: uptake pattern (grades 1–4), exact location of the focal uptake, extent of the uptake, intensity of the uptake. Whole-body imaging describe extracardiac locations with increased [^18^F]FDG uptake. Abnormalities on low dose or CTA scan should be described. Comparison with prior [^18^F]FDG -PET scan, if scan is performed in the context of assessment therapy response, then extent and intensity should be compared to prior scan: increase in uptake, equal uptake, decrease in uptake. Comparison with other imaging modalities, echocardiography, MRI, and with specific PET amyloid compounds that have been evaluated in patients with AL and ATTR cardiac amyloidosis. For [^11^C]-PIB, [^18^F]-florbetapir, [^18^F]-florbetaben, and [^18^F]flutemetamol, see paragraph Specific PET display for analysis and Table 1.

### Atherosclerosis (clinical research)

Cardiovascular PET imaging has the potential to provide complementary information to the anatomical assessment of atherosclerotic plaques offered by computed tomography, relating to disease activity. [^18^F]FDG is still a focus of interest, especially in larger vessels such as the aorta and carotids, despite its well-known limitations in the imaging of atherosclerosis. In an effort to overcome some of those limitations and in order to standardize arterial PET imaging and thereby facilitate multicenter atherosclerosis PET studies, a position paper was published by the Cardiovascular Committee of the European Association of Nuclear Medicine (EANM) in 2016 (18). This position paper addresses critical issues regarding patient preparation, the [^18^F]FDG PET imaging protocol, and data analysis. This document served as the basis for the information in this procedural recommendation. [^18^F]FDG faces several limitations with regard to atherosclerosis imaging particularly in the coronary arteries. Several new PET tracers have been developed or are under development to overcome those limitations. In addition, these tracers might provide new pathological insights and allow evaluation of new targets for future diagnostic and therapeutic approaches.

#### Study indication

- Measuring disease activity in atherosclerosis
- Patient risk stratification for recurrent ischemic events
- Monitoring treatment response

#### Image analysis and interpretation of large vessel [^18^F]-FDG PET

Vascular, uptake pattern, diffuse or focal [^18^F]FDG, exact location, distribution of the uptake/vascular segments, intensity (vascular scoring 0–3 against the liver). Focal uptake in the aorta is more consistent with atherosclerotic activity as compared to the diffuse high intensity uptake observed in large vessel vasculitis. For the quantification of [^18^F]FDG uptake in atherosclerotic plaques, application of TBR analysis instead of SUV is recommended, using the (venous) blood pool activity as background. The latter should be measured in a consistent fashion. TBR values provide a ratio between two measurements thereby limiting the effects on signal quantification of errors in patient weight, the applied activity of the injected radiotracer and the acquisition time point. Whole-body imaging describe extravascular locations with increased [^18^F]FDG uptake. Abnormalities on low dose or CTA scan should be described such as the atherosclerotic plaque burden, presence of obstructive stenoses and plaque composition and shape for the identification of high risk plaques. As described, CTA is recommended when assessing the coronary arteries. Comparison with prior [^18^F]FDG PET scan: if scan is performed to assess response to therapy, then extent and intensity should be compared to prior scans: increase in uptake, equal uptake, decrease in uptake. An advantage of [^18^F]FDG PET imaging is that changes in uptake are detectable as early as 3–4 months after initiation of drug treatment, whereas morphological changes, for instance in plaque volume, may only be visual later on during the disease process (12–24 months). [^18^F]FDG has therefore been used as an endpoint in clinical trials investigating novel atherosclerotic treatments. Comparison with other imaging modalities: CTA, MRA, and alternative PET radiopharmaceuticals, such as [^18^F]NaF and [^68^Ga]DOTA conjugated peptides to SSRs. The target-to-background uptake ratio of [^18^F]NaF exceeds that of [^18^F]FDG and may be more promising for future clinical applications particularly in the coronary arteries (85). The maximum arterial radioactivity concentration of [^68^Ga]DOTA conjugated can be normalized by mean blood pool activity in the superior vena cava (maximum tissue-to-blood ratio [TBRmax], and demonstrated a better power to discriminate high-risk versus low-risk coronary lesions than [^18^F]FDG (3). Time-of-flight and point-spread-function may potentially improve the detection of atherosclerosis activity on PET/CT (86). For CTA analysis and interpretation, see Table 2.

### Myocardial innervation (clinical research)

Novel methods and different PET ligands have been developed to measure presynaptic and postsynaptic function of the cardiac neuronal system. There is also an increasing need for identification of new and refinement of existing methods for noninvasive risk stratification in patients with heart failure, particularly to help identify patients at risk for ventricular tachyarrhythmia’s and sudden cardiac death (87, 88). The most commonly used PET tracer for imaging presynaptic sympathetic function is [^11^C]mHED, which is also a norepinephrine analogue. Standardization is needed for PET myocardial innervation imaging, including patient preparation before the PET procedure such as medication withdrawal (89), as described in the Guidelines on [^123^I]mIBG (30).

#### Study indication

- Assessing severity and prognosis of heart failure
- Assessing risk of ventricular arrhythmias or sudden cardiac death
- Clinical trial phase

#### Image analysis and interpretation

Originally, kinetic analysis of [^11^C]mHED data was performed according to compartment models, which requires good understanding of the different compartments to which the tracer is distributed. Different compartments are connected by rate constants, which describe the exchange of tracer between them, using blood sampling (arterial preferable) as input and reference. As an alternative to these complex procedures, [^11^C]mHED uptake is now commonly quantified through a retention index, which is defined as the ratio of the activity in the myocardium in the final image of a 40- or 60-min dynamic sequence to the integral of the image-derived arterial blood time–activity curve. A volume of interest (VOI) for the input function is placed in the basal plane; the VOI for tissue curves is place within the left ventricular wall. Acquisition of [^11^C]mHED data can also be performed using ECG gating, for simultaneous analysis of LV volumes and function. Distribution of [^11^C]mHED throughout the left ventricular myocardium in healthy normal individuals is regionally homogeneous around the wall with high uptake in all myocardial segments. Visual interpretation of polar maps, analogue to and alongside those of myocardial perfusion, provides information for global innervation status. Generally, perfusion PET images are also acquired in conjunction with the innervation study to evaluate perfusion-innervation mismatch areas. With the 17-segment AHA model, detailed information about perfusion–innervation relationships can be obtained (40, 88). Mean [^11^C]mHED retention can be determined for those areas with both normal perfusion (> 80% of the maximum myocardial blood flow) and innervation (> 75% of the segment with maximum [^11^C]mHED retention), areas with a mismatch pattern: normal perfusion and decreased cardiac sympathetic innervation (< 75% of the segment with maximum [^11^C]mHED retention), and both abnormal perfusion (< 80% of the maximum myocardial blood flow) and innervation (< 75% of the segment with maximum [^11^C]mHED retention). Comparison with other imaging modalities: CTA, MRA.

## PET/CT pitfalls

PET/CT imaging is associated with several potential pitfalls. These commonly include inadequate patient preparation, motion scatter, and PET/CT mismatch artifacts (90). See Table 5 for more details among pitfalls in 4Is PET/CT imaging.

**Table 5:** Pitfalls in PET/CT imaging of inflammatory-, infective-, infiltrative-, and innervation (4Is)-related cardiovascular diseases

Patient preparation	Acquisition	Reconstruction	Reading
Elevated blood glucose resulting in low quality [^18^F]FDG PET	Motion artifacts	Mismatch fusion PET and CT	False positive uptake surrounding
Diet not followed and no suppression of myocardial uptake resulting in low quality [^18^F]FDG PET	Metal/scatter/beam-hardening/highly calcification artifacts	Truncation	Pathological conditions, such as thrombi, tumor, (FP)
Use of interfering drugs such as antibiotics, steroids resulting in reduced sensitivity of [^18^F]FDG PET Drug interfering with cardiac presynaptic sympathetic tracers resulting in reduced sensitivity of [^11^C]mHED PET	Arrhythmias	No standardization	Short interval between surgery and imaging
	No standardization		No standardization

## PET/MR procedurals in 4Is

So far, the evidence for using PET/MR imaging in 4Is is very limited, but some overview papers have been published in the field of cardiovascular diseases (12, 91–93). For the PET acquisition and reconstruction in PET/MR, recommendations are identical to PET/CT imaging. Currently commercially available Dixon-based MR-based attenuation correction methods using four tissue class segmentation are preferred for [^18^F]FDG quantification (94) and have been shown to yield quantitatively comparable results as PET/CT for perfusion imaging with [^15^O]water (95). In general, the use of attenuation correction methods incorporating bone should be encouraged when they become available (96). When available, the use of free-breathing radial VIBE sequences for AC is encouraged since these AC sequences appear to reduce breathing and motion artifacts (97). Particular caution is warranted in case of MR-compatible, implanted material such as stents or sternal wires, as these can lead to incorrect attenuation maps and thus to incorrect attenuation-corrected PET data (98). In addition, endocarditis and device infection is not a major application for PET/MR due to the interaction between ferromagnetic material and the magnetic field. Another potential source of error is the attenuation of the PET signal by MR equipment such as radiofrequency coils (99). This can be overcome by using predefined attenuation map templates for, e.g., the patient table and nonflexible coils. This approach is not feasible for flexible coils, however the flexible RF coils are designed PET-transparent and have shown to cause an attenuation bias of only 3–5% (99). It should be emphasized that not all of the possible corrections of PET attenuation are implemented in clinical routine. In summary, however, the correction of PET/MR attenuation can be considered accurate and robust. While correction of PET data for motion based on MR data represents a promising approach, it is not yet routinely implemented.

Major fields of application of PET/MR:

- Cardiac sarcoidosis could be a major field of application for PET/MR (100, 101). Myocardial perfusion, scars, detailed morphological analysis, and functional parameters can be assessed by MR while PET depicts actively diseased myocardial areas with high sensitivity and is very well suited for assessing the course of the disease (therapy response/guidance/monitoring) (102, 103).
- LVV including giant cell arteritis and Takayasu arteritis. The few existing studies indicate that PET/MR is suitable to determine the extent of the disease and to determine disease activity (104, 105).
- Atherosclerosis: MRI allows detailed morphological characterization of aortic and carotid plaques, including assessments of the fibrous cap or the lipid core, while PET is capable of imaging the inflammatory activity or, more generally, the vulnerability. The most commonly used tracer is [^18^F]FDG (106, 107), but initial work on other tracers such as [^18^F]NaF (107) or other targets such as the chemokine receptor 4 (108) or amyloid deposits (109) are also underway.
- Cardiac amyloidosis: MRI can be used to confirm the suspicion of cardiac involvement in amyloidosis; PET allows specific imaging of amyloid deposits using amyloid-specific tracers. A PET/MRI study on the use of [^18^F]NaF in cardiac amyloidosis has shown the feasibility of this tracer providing similar information to Tc-99-m labeled bisphosphonates (84, 110).
- Myocardial innervation imaging, no relevant applications are currently envisioned using PET/MRI.

## The 4Is-team

A multidisciplinary team approach has been proposed as the model in oncology in many hospitals and medical centers. More recently, this approach has been extended to cardiology with the successful introduction of the heart valve team for the assessment of patients being considered for transcutaneous aortic valve implantation (111). In the field of infective endocarditis (IE), a multidisciplinary approach for evaluating patients with IE has also been introduced in order to improve management and outcome. This example can be extended to all complex disease states including the 4Is. We would therefore advocate creation of a 4Is-team of experts to improve clinical assessment of decision making for these complex patients. To be effective, the structure of a 4Is team has to be modeled on the local health systems, including cultural and socioeconomic aspects. Its success is contingent upon knowledge of one’s own area of expertise as well as that of the team members, flexibility of roles, and comfort and skills in supplying and receiving interdisciplinary education. To promote effective collaboration, the team must address issues of group dynamics, including clarification of individual roles, team unity, communication, and patterns of decision-making and leadership. The clinical imager plays an active role in the teamwork program and in the global educational planning, developing “capabilities” and “competencies”, core skills, knowledge and attitudes to facilitate interspecialist communications. The challenge of the clinical imager within the 4Is team is to establish a new professional perspective: a new vision of the imager, no longer thinking as an individual, but rather as an integral player and contributor to the team, translating the image content into clinical planning and a decision-making process that enhance the quality of patient care.

## Conclusions

With this document, we provided a standard for PET/CT imaging in inflammatory, infective, infiltrative, and innervation dysfunctional (4Is) cardiovascular diseases. It can be applied in clinical practice and integrated in (multicenter) clinical trials for optimal procedural standardization. 4Is-related cardiovascular diseases are generally complex and often require wide ranging expertise and a multidisciplinary approach for optimal diagnosis and management. New PET 4Is radiopharmaceuticals beyond [^18^F]FDG are available, but are currently mainly in the clinical research phase. Further clinical evaluation of the most promising PET tracers is warranted before their implementation in routine clinical practice.

## References

[1] Jamar F, Buscombe J, Chiti A, Christian PE, Delbeke D, Donohoe KJ (2013). EANM/SNMMI guideline for 18F-FDG use in inflammation and infection. J Nucl Med.

[2] Joshi NV, Vesey AT, Williams MC, Shah AS, Calvert PA, Craighead FH (2014). 18F-fluoride positron emission tomography for identification of ruptured and high-risk coronary atherosclerotic plaques: a prospective clinical trial. Lancet..

[3] Tarkin JM, Joshi FR, Evans NR, Chowdhury MM, Figg NL, Shah AV (2017). Detection of atherosclerotic inflammation by (68)Ga-DOTATATE PET compared to [(18)F]FDG PET imaging. J Am Coll Cardiol.

[4] Minoshima S, Drzezga AE, Barthel H, Bohnen N, Djekidel M, Lewis DH (2016). SNMMI procedure standard/EANM practice guideline for amyloid PET imaging of the brain 1.0. J Nucl Med.

[5] Dorbala S, Ando Y, Bokhari S, Dispenzieri A, Falk RH, Ferrari VA (2019). ASNC/AHA/ASE/EANM/HFSA/ISA/SCMR/SNMMI expert consensus recommendations for multimodality imaging in cardiac amyloidosis: part 1 of 2-evidence base and standardized methods of imaging. J Nucl Cardiol.

[6] Dorbala S, Ando Y, Bokhari S, Dispenzieri A, Falk RH, Ferrari VA, et al. ASNC/AHA/ASE/EANM/HFSA/ISA/SCMR/SNMMI expert consensus recommendations for multimodality imaging in cardiac amyloidosis: part 2 of 2-diagnostic criteria and appropriate utilization. J Nucl Cardiol. 2019;29. 10.1007/s12350-019-01761-5 31468377

[7] Schwaiger M, Kalff V, Rosenspire K, Haka MS, Molina E, Hutchins GD (1990). Noninvasive evaluation of sympathetic nervous system in human heart by positron emission tomography. Circulation..

[8] Boellaard R, Delgado-Bolton R, Oyen WJ, Giammarile F, Tatsch K, Eschner W (2015). FDG PET/CT: EANM procedure guidelines for tumour imaging: version 2.0. Eur J Nucl Med Mol Imaging.

[9] Aide N, Lasnon C, Veit-Haibach P, Sera T, Sattler B, Boellaard R (2017). EANM/EARL harmonization strategies in PET quantification: from daily practice to multicentre oncological studies. Eur J Nucl Med Mol Imaging.

[10] Busemann Sokole E, Plachcinska A, Britten A (2010). EANM physics committee. Acceptance testing for nuclear medicine instrumentation. Eur J Nucl Med Mol Imaging.

[11] Erba PA, Lancellotti P, Vilacosta I, Gaemperli O, Rouzet F, Hacker M (2018). Recommendations on nuclear and multimodality imaging in IE and CIED infections. Eur J Nucl Med Mol Imaging.

[12] Habib G, Bucciarelli-Ducci C, Caforio ALP, Cardim N, Charron P, Cosyns B (2017). Multimodality imaging in restrictive cardiomyopathies: an EACVI expert consensus document in collaboration with the “Working Group on myocardial and pericardial diseases” of the European Society of Cardiology Endorsed by the Indian academy of echocardiography. Eur Heart J Cardiovasc Imaging.

[13] Habib G, Lancellotti P, Antunes MJ, Bongiorni MG, Casalta JP, Del Zotti F (2015). 2015 ESC guidelines for the management of infective endocarditis: the Task Force for the Management of Infective Endocarditis of the European Society of Cardiology (ESC). Endorsed by: European Association for Cardio-Thoracic Surgery (EACTS), the European Association of Nuclear Medicine (EANM). Eur Heart J.

[14] Chakfe N, Diener H, Lejay A, Assadian O, Berard X, Caillon J (2020). Editor’s choice - European Society for Vascular Surgery (ESVS) 2020 Clinical practice guidelines on the management of vascular graft and endograft infections. Eur J Vasc Endovasc Surg.

[15] Slart RHJ, Glaudemans WJM, Lancellotti P, Hyafil F, Blankstein R, Schwartz RG (2018). a joint procedural position statement on imaging in cardiac sarcoidosis: from the Cardiovascular and Inflammation & Infection Committees of the European Association of Nuclear Medicine, the European Association of Cardiovascular Imaging, and the American Society of Nuclear Cardiology. J Nucl Cardiol.

[16] Slart RHJA, writing group, reviewer group, members of EANM cardiovascular, members of EANM Infection & Inflammation, members of committees, SNMMI cardiovascular (2018). FDG-PET/CT(A) imaging in large vessel vasculitis and polymyalgia rheumatica: joint procedural recommendation of the EANM, SNMMI, and the PET Interest Group (PIG), and endorsed by the ASNC. Eur J Nucl Med Mol Imaging.

[17] Dejaco C, Ramiro S, Duftner C, Besson FL, Bley TA, Blockmans D (2018). EULAR recommendations for the use of imaging in large vessel vasculitis in clinical practice. Ann Rheum Dis.

[18] Bucerius J, Hyafil F, Verberne HJ, Slart RH, Lindner O, Sciagra R (2016). Position paper of the Cardiovascular Committee of the European Association of Nuclear Medicine (EANM) on PET imaging of atherosclerosis. Eur J Nucl Med Mol Imaging.

[19] Slart RHJA, Tio RA, Elsinga PH, Schwaiger M (2015). Autonomic innervation of the heart. Role of molecular imaging.

[20] Dorbala S, Vangala D, Semer J, Strader C, Bruyere JR, Di Carli MF (2014). Imaging cardiac amyloidosis: a pilot study using (1)(8)F-florbetapir positron emission tomography. Eur J Nucl Med Mol Imaging.

[21] Kero T, Sorensen J, Antoni G, Wilking H, Carlson K, Vedin O, et al. Quantification of (11)C-PIB kinetics in cardiac amyloidosis. J Nucl Cardiol. 2018;23. 10.1007/s12350-018-1349-x PMC7326793 30039218

[22] Dorbala S, Di Carli MF, Delbeke D, Abbara S, DePuey EG, Dilsizian V (2013). SNMMI/ASNC/SCCT guideline for cardiac SPECT/CT and PET/CT 1.0. J Nucl Med.

[23] Osborne MT, Hulten EA, Murthy VL, Skali H, Taqueti VR, Dorbala S (2017). Patient preparation for cardiac fluorine-18 fluorodeoxyglucose positron emission tomography imaging of inflammation. J Nucl Cardiol.

[24] Rabkin Z, Israel O, Keidar Z (2010). Do hyperglycemia and diabetes affect the incidence of false-negative 18F-FDG PET/CT studies in patients evaluated for infection or inflammation and cancer? A Comparative analysis. J Nucl Med.

[25] Scholtens AM, van Aarnhem EE, Budde RP (2015). Effect of antibiotics on FDG-PET/CT imaging of prosthetic heart valve endocarditis. Eur Heart J Cardiovasc Imaging.

[26] Swart LE, Gomes A, Scholtens AM, Sinha B, Tanis W, Lam, M G E H, et al. improving the diagnostic performance of (18)F-Fluorodeoxyglucose positron-emission tomography/computed tomography in prosthetic heart valve endocarditis. Circulation. 2018;138(14):1412–1427. 10.1161/CIRCULATIONAHA.118.035032 30018167

[27] Nielsen BD, Gormsen LC, Hansen IT, Keller KK, Therkildsen P, Hauge EM (2018). Three days of high-dose glucocorticoid treatment attenuates large-vessel 18F-FDG uptake in large-vessel giant cell arteritis but with a limited impact on diagnostic accuracy. Eur J Nucl Med Mol Imaging.

[28] Minamimoto R, Takahashi N, Inoue T (2007). FDG-PET of patients with suspected renal failure: standardized uptake values in normal tissues. Ann Nucl Med.

[29] Bozkurt MF, Virgolini I, Balogova S, Beheshti M, Rubello D, Decristoforo C (2017). Guideline for PET/CT imaging of neuroendocrine neoplasms with (68)Ga-DOTA-conjugated somatostatin receptor targeting peptides and (18)F-DOPA. Eur J Nucl Med Mol Imaging.

[30] Flotats A, Carrio I, Agostini D, Le Guludec D, Marcassa C, Schafers M (2010). Proposal for standardization of 123I-metaiodobenzylguanidine (MIBG) cardiac sympathetic imaging by the EANM Cardiovascular Committee and the European Council of Nuclear Cardiology. Eur J Nucl Med Mol Imaging.

[31] Rosenblum JS, Quinn KA, Rimland CA, Mehta NN, Ahlman MA, Grayson PC (2019). Clinical factors associated with time-specific distribution of 18F-fluorodeoxyglucose in large-vessel vasculitis. Sci Rep.

[32] Blomberg BA, Bashyam A, Ramachandran A, Gholami S, Houshmand S, Salavati A (2015). Quantifying [(1)(8)F]fluorodeoxyglucose uptake in the arterial wall: the effects of dual time-point imaging and partial volume effect correction. Eur J Nucl Med Mol Imaging.

[33] Scholtens AM, Swart LE, Verberne HJ, Budde RPJ, Lam MGEH (2018). Dual-time-point FDG PET/CT imaging in prosthetic heart valve endocarditis. J Nucl Cardiol.

[34] Nielsen BD, Hansen IT, Kramer S, Haraldsen A, Hjorthaug K, Bogsrud TV (2019). Simple dichotomous assessment of cranial artery inflammation by conventional 18F-FDG PET/CT shows high accuracy for the diagnosis of giant cell arteritis: a case-control study. Eur J Nucl Med Mol Imaging.

[35] Boursier C, Duval X, Bourdon A, Imbert L, Mahida B FAU -valier, Elodie, Chevalier E, et al. ECG-gated cardiac FDG PET acquisitions significantly improve detectability of infective endocarditis. JACC Cardiovasc Imaging. 2020 10.1016/j.jcmg.2020.06.036 32828775

[36] Kero T, Lindsjo L, Sorensen J, Lubberink M (2016). Accurate analysis and visualization of cardiac (11)C-PIB uptake in amyloidosis with semiautomatic software. J Nucl Cardiol.

[37] Rosengren S, Skibsted Clemmensen T, Tolbod L, Granstam SO, Eiskjær H, Wikström G (2020). Diagnostic accuracy of [11C]PIB positron emission tomography for detection of cardiac amyloidosis. JACC Cardiovasc Imaging.

[38] Dietemann S, Nkoulou R (2019). Amyloid PET imaging in cardiac amyloidosis: a pilot study using (18)F-flutemetamol positron emission tomography. Ann Nucl Med.

[39] Noordzij W, Slart RH (2015). PET imaging of the autonomic myocardial function: methods and interpretation. Clin Transl Imaging..

[40] Noordzij W, Elvan A, Demirel F, Jager PL, Tio RA, Slart RH (2018). Sympathetic denervation in patients with ischemic cardiomyopathy and risk on ventricular tachy-arrhythmias. A pilot study. Q J Nucl Med Mol Imaging.

[41] Kim JM, Lee ES, Park KY, Seok JW, Kwon OS (2019). Comparison of [(18)F]-FDG and [(18)F]-NaF positron emission tomography on culprit carotid atherosclerosis: a prospective study. JACC Cardiovasc Imaging.

[42] Moss AJ, Doris MK, Andrews JPM, Bing R, Daghem M, van Beek EJR (2019). Molecular coronary plaque imaging using (18)F-fluoride. Circ Cardiovasc Imaging.

[43] Rubeaux M, Joshi NV, Dweck MR, Fletcher A, Motwani M, Thomson LE (2016). Motion correction of 18F-NaF PET for imaging coronary atherosclerotic plaques. J Nucl Med.

[44] Cartlidge TRG, Doris MK, Sellers SL, Pawade TA, White AC, Pessotto R (2019). Detection and prediction of bioprosthetic aortic valve degeneration. J Am Coll Cardiol.

[45] Vesey AT, Jenkins WS, Irkle A, Moss A, Sng G, Forsythe RO, et al. (18)F-fluoride and (18)F-fluorodeoxyglucose positron emission tomography after transient ischemic attack or minor ischemic stroke: case-control study. Circ Cardiovasc Imaging. 2017;10(3):10.1161/CIRCIMAGING.116.004976. 10.1161/CIRCIMAGING.116.004976 PMC5367506 28292859

[46] Pawade TA, Cartlidge TR, Jenkins WS, Adamson PD, Robson P, Lucatelli C, et al. Optimization and reproducibility of aortic valve 18f-fluoride positron emission tomography in patients with aortic stenosis. Circ Cardiovasc Imaging. 2016;9(10):10.1161/CIRCIMAGING.116.005131. 10.1161/CIRCIMAGING.116.005131 PMC5068186 27733431

[47] Schaefferkoetter J, Ouyang J, Rakvongthai Y, Nappi C, El Fakhri G (2014). Effect of time-of-flight and point spread function modeling on detectability of myocardial defects in PET. Med Phys.

[48] Lindstrom E, Sundin A, Trampal C, Lindsjo L, Ilan E, Danfors T (2018). Evaluation of penalized-likelihood estimation reconstruction on a digital time-of-flight PET/CT scanner for (18)F-FDG whole-body examinations. J Nucl Med.

[49] Antoni G, Lubberink M, Estrada S, Axelsson J, Carlson K, Lindsjo L (2013). In vivo visualization of amyloid deposits in the heart with 11C-PIB and PET. J Nucl Med.

[50] Kwiecinski J, Cadet S, Daghem M, Lassen ML, Dey D, Dweck MR, et al. Whole-vessel coronary (18)F-sodium fluoride PET for assessment of the global coronary microcalcification burden. Eur J Nucl Med Mol Imaging. 2020. 10.1007/s00259-019-04667-z PMC7271818 31897586

[51] Nijssen EC, Nelemans PJ, Rennenberg RJ, van der Molen, A J, van Ommen GV, Wildberger JE. Impact on clinical practice of updated guidelines on iodinated contrast material: CINART. Eur Radiol. 2020. 10.1007/s00330-020-06719-7 PMC7305084 32107605

[52] Pizzi MN, Roque A, Fernandez-Hidalgo N, Cuellar-Calabria H, Ferreira-Gonzalez I, Gonzalez-Alujas MT (2015). Improving the diagnosis of infective endocarditis in prosthetic valves and Intracardiac devices with 18F-fluordeoxyglucose positron emission tomography/computed tomography angiography: initial results at an infective endocarditis referral center. Circulation..

[53] Roque A, Pizzi MN, Cuellar-Calabria H, Aguade-Bruix S (2017). (18)F-FDG-PET/CT angiography for the diagnosis of infective endocarditis. Curr Cardiol Rep.

[54] Quinn KA, Grayson PC (2019). The role of vascular imaging to advance clinical care and research in large-vessel vasculitis. Curr Treatm Opt Rheumatol.

[55] Menke J, Unterberg-Buchwald C, Staab W, Sohns JM, Seif Amir Hosseini A, Schwarz A (2013). Head-to-head comparison of prospectively triggered vs retrospectively gated coronary computed tomography angiography: meta-analysis of diagnostic accuracy, image quality, and radiation dose. Am Heart J.

[56] Knuuti J, Wijns W, Saraste A, Capodanno D, Barbato E, Funck-Brentano C (2020). 2019 ESC guidelines for the diagnosis and management of chronic coronary syndromes. Eur Heart J.

[57] Newby DE, Adamson PD, Berry C, Boon NA, Dweck MR, SCOT-HEART Investigators (2018). Coronary CT angiography and 5-year risk of myocardial infarction. N Engl J Med.

[58] Swart LE, Scholtens AM, Tanis W, Nieman K, Bogers JJC, Verzijlbergen FJ (2018). 18F-fluorodeoxyglucose positron emission/computed tomography and computed tomography angiography in prosthetic heart valve endocarditis: from guidelines to clinical practice. Eur Heart J.

[59] Habib G, Erba PA, Iung B, Donal E, Cosyns B, Laroche C (2019). Clinical presentation, aetiology and outcome of infective endocarditis. Results of the ESC-EORP EURO-ENDO (European infective endocarditis) registry: a prospective cohort study. Eur Heart J.

[60] Hyafil F, Rouzet F, Le Guludec D (2017). Nuclear imaging for patients with a suspicion of infective endocarditis: be part of the team!. J Nucl Cardiol.

[61] Gomes A, Glaudemans WJM, Touw DJ, van Melle JP, Willems TP, Maass AH (2017). Diagnostic value of imaging in infective endocarditis: a systematic review. Lancet Infect Dis.

[62] Cantoni C, Solline M, Green R, Berchiolli R, Lazzeri E, Mannarino T (2018). Comprehensive meta-analysis on [18F] FDG PET/CT and radiolabelled leukocyte SPECT–SPECT/CT imaging in infectious endocarditis and cardiovascular implantable electronic device infections. Clin Transl Imaging.

[63] Pelletier-Galarneau M, Abikhzer G, Harel F, Dilsizian V (2020). Detection of native and prosthetic valve endocarditis: incremental attributes of functional FDG PET/CT over morphologic imaging. Curr Cardiol Rep.

[64] de Camargo RA, Sommer Bitencourt M, Meneghetti JC, Soares J, Goncalves LFT, Buchpiguel CA (2020). The role of 18F-fluorodeoxyglucose positron emission tomography/computed tomography in the diagnosis of left-sided endocarditis: native vs prosthetic valves endocarditis. Clin Infect Dis.

[65] Klug D, Wallet F, Lacroix D, Marquie C, Kouakam C, Kacet S (2004). Local symptoms at the site of pacemaker implantation indicate latent systemic infection. Heart..

[66] Bongiorni MG, Burri H, Deharo JC, Starck C, Kennergren C, Saghy L (2018). 2018 EHRA expert consensus statement on lead extraction: recommendations on definitions, endpoints, research trial design, and data collection requirements for clinical scientific studies and registries: endorsed by APHRS/HRS/LAHRS. Europace..

[67] Sarrazin JF, Philippon F, Tessier M, Guimond J, Molin F, Champagne J (2012). Usefulness of fluorine-18 positron emission tomography/computed tomography for identification of cardiovascular implantable electronic device infections. J Am Coll Cardiol.

[68] Leccisotti L, Perna F, Lago M, Leo M, Stefanelli A, Calcagni ML (2014). Cardiovascular implantable electronic device infection: delayed vs standard FDG PET-CT imaging. J Nucl Cardiol.

[69] Blomstrom-Lundqvist C, Traykov V, Erba PA, Burri H, Nielsen JC, Bongiorni MG (2020). European Heart Rhythm Association (EHRA) international consensus document on how to prevent, diagnose, and treat cardiac implantable electronic device infections-endorsed by the Heart Rhythm Society (HRS), the Asia Pacific Heart Rhythm Society (APHRS), the Latin American Heart Rhythm Society (LAHRS), International Society for Cardiovascular Infectious Diseases (ISCVID) and the European Society of Clinical Microbiology and Infectious Diseases (ESCMID) in collaboration with the European Association for Cardio-Thoracic Surgery (EACTS). Europace.

[70] Memmott MJ, James J, Armstrong IS, Tout D, Ahmed F (2016). The performance of quantitation methods in the evaluation of cardiac implantable electronic device (CIED) infection: a technical review. J Nucl Cardiol.

[71] Hyafil F, Rouzet F, Benali K (2019). FDG-PET for the detection of infection in left ventricle assist device: is there light at the end of the tunnel?. J Nucl Cardiol.

[72] de Vaugelade C, Mesguich C, Nubret K, Camou F, Greib C, Dournes G (2019). Infections in patients using ventricular-assist devices: comparison of the diagnostic performance of (18)F-FDG PET/CT scan and leucocyte-labeled scintigraphy. J Nucl Cardiol.

[73] Valentine RJ (2001). Diagnosis and management of aortic graft infection. Semin Vasc Surg.

[74] Saleem BR, Berger P, Vaartjes I, de Keizer B, Vonken EJ, Slart RH (2015). Modest utility of quantitative measures in (18)F-fluorodeoxyglucose positron emission tomography scanning for the diagnosis of aortic prosthetic graft infection. J Vasc Surg.

[75] Keidar Z, Pirmisashvili N, Leiderman M, Nitecki S, Israel O (2014). 18F-FDG uptake in noninfected prosthetic vascular grafts: incidence, patterns, and changes over time. J Nucl Med.

[76] Lauri C, Iezz R, Rossi M, Tinelli G, Sica S, Signore A, et al. Imaging modalities for the diagnosis of vascular graft infections: a consensus paper amongst different specialists. J Clin Med. 2020;9(5):10.3390/jcm9051510. 10.3390/jcm9051510 PMC7290746 32429584

[77] Birnie DH, Sauer WH, Bogun F, Cooper JM, Culver DA, Duvernoy CS (2014). HRS expert consensus statement on the diagnosis and management of arrhythmias associated with cardiac sarcoidosis. Heart Rhythm.

[78] Dweck MR, Abgral R, Trivieri MG, Robson PM, Karakatsanis N, Mani V (2018). Hybrid magnetic resonance imaging and positron emission tomography with fluorodeoxyglucose to diagnose active cardiac Sarcoidosis. JACC Cardiovasc Imaging.

[79] Flores RJ, Flaherty KR, Jin Z, Bokhari S. The prognostic value of quantitating and localizing F-18 FDG uptake in cardiac sarcoidosis. J Nucl Cardiol. 2018. 10.1007/s12350-018-01504-y 30421379

[80] Gormsen LC, Haraldsen A, Kramer S, Dias AH, Kim WY, Borghammer P (2016). A dual tracer (68)Ga-DOTANOC PET/CT and (18)F-FDG PET/CT pilot study for detection of cardiac sarcoidosis. EJNMMI Res.

[81] Misra DP, Shenoy SN (2017). Cardiac involvement in primary systemic vasculitis and potential drug therapies to reduce cardiovascular risk. Rheumatol Int.

[82] Glaudemans AW, Slart RH, Noordzij W, Dierckx RA, Hazenberg BP (2013). Utility of 18F-FDG PET(/CT) in patients with systemic and localized amyloidosis. Eur J Nucl Med Mol Imaging.

[83] Kircher M, Ihne S, Brumberg J, Morbach C, Knop S, Kortum KM (2019). Detection of cardiac amyloidosis with (18)F-Florbetaben-PET/CT in comparison to echocardiography, cardiac MRI and DPD-scintigraphy. Eur J Nucl Med Mol Imaging.

[84] Abulizi M, Sifaoui I, Wuliya-Gariepy M, Kharoubi M, Israel JM, Emsen B, et al. (18)F-sodium fluoride PET/MRI myocardial imaging in patients with suspected cardiac amyloidosis. J Nucl Cardiol. 2019. 10.1007/s12350-019-01885-8 31512197

[85] McKenney-Drake ML, Moghbel MC, Paydary K, Alloosh M, Houshmand S, Moe S (2018). (18)F-NaF and (18)F-FDG as molecular probes in the evaluation of atherosclerosis. Eur J Nucl Med Mol Imaging.

[86] van der Vos CS, Koopman D, Rijnsdorp S, Arends AJ, Boellaard R, van Dalen JA (2017). Quantification, improvement, and harmonization of small lesion detection with state-of-the-art PET. Eur J Nucl Med Mol Imaging.

[87] Narula J, Gerson M, Thomas GS, Cerqueira MD, Jacobson AF (2015). (1)(2)(3)I-MIBG imaging for prediction of mortality and potentially fatal events in heart failure: the ADMIRE-HFX study. J Nucl Med.

[88] Fallavollita JA, Heavey BM, Luisi AJ, Michalek SM, Baldwa S, Mashtare TL (2014). Regional myocardial sympathetic denervation predicts the risk of sudden cardiac arrest in ischemic cardiomyopathy. J Am Coll Cardiol.

[89] Jacobson AF, Travin MI (2015). Impact of medications on mIBG uptake, with specific attention to the heart: comprehensive review of the literature. J Nucl Cardiol.

[90] Glaudemans AW, Israel O, Slart RH (2015). Pitfalls and limitations of radionuclide and hybrid imaging in infection and inflammation. Semin Nucl Med.

[91] Nensa F, Kloth J, Tezgah E, Poeppel TD, Heusch P, Goebel J (2018). Feasibility of FDG-PET in myocarditis: comparison to CMR using integrated PET/MRI. J Nucl Cardiol.

[92] Nazir MS, Ismail TF, Reyes E, Chiribiri A, Kaufmann PA, Plein S (2018). Hybrid positron emission tomography-magnetic resonance of the heart: current state of the art and future applications. Eur Heart J Cardiovasc Imaging.

[93] Robson PM, Dey D, Newby DE, Berman D, Li D, Fayad ZA (2017). MR/PET Imaging of the Cardiovascular System. JACC Cardiovasc Imaging.

[94] Robson PM, Vergani V, Benkert T, Trivieri MG, Karakatsanis NA, Abgral R, et al. Assessing the qualitative and quantitative impacts of simple two-class vs multiple tissue-class MR-based attenuation correction for cardiac PET/MR. J Nucl Cardiol. 2020. 10.1007/s12350-019-02002-5 PMC7329599 31898004

[95] Kero T, Nordstrom J, Harms HJ, Sorensen J, Ahlstrom H, Lubberink M (2017). Quantitative myocardial blood flow imaging with integrated time-of-flight PET-MR. EJNMMI Phys.

[96] Lindemann ME, Nensa F, Quick HH (2019). Impact of improved attenuation correction on 18F-FDG PET/MR hybrid imaging of the heart. PLoS One.

[97] Andrews JPM, MacNaught G, Moss AJ, Doris MK, Pawade T, Adamson PD, et al. Cardiovascular (18)F-fluoride positron emission tomography-magnetic resonance imaging: a comparison study. J Nucl Cardiol. 2019. 10.1007/s12350-019-01962-y PMC8616877 31792913

[98] Lassen ML, Rasul S, Beitzke D, Stelzmuller ME, Cal-Gonzalez J, Hacker M (2019). Assessment of attenuation correction for myocardial PET imaging using combined PET/MRI. J Nucl Cardiol.

[99] Paulus DH, Tellmann L, Quick HH (2013). Towards improved hardware component attenuation correction in PET/MR hybrid imaging. Phys Med Biol.

[100] Rischpler C, Nekolla SG, Kunze KP, Schwaiger M (2015). PET/MRI of the heart. Semin Nucl Med.

[101] Nensa F, Bamberg F, Rischpler C, Menezes L, Poeppel TD, la Fougere C (2018). Hybrid cardiac imaging using PET/MRI: a joint position statement by the European Society of Cardiovascular Radiology (ESCR) and the European Association of Nuclear Medicine (EANM). Eur Radiol.

[102] Schneider S, Batrice A, Rischpler C, Eiber M, Ibrahim T, Nekolla SG (2014). Utility of multimodal cardiac imaging with PET/MRI in cardiac sarcoidosis: implications for diagnosis, monitoring and treatment. Eur Heart J.

[103] Nensa F, Tezgah E, Poeppel T, Nassenstein K, Schlosser T (2015). Diagnosis and treatment response evaluation of cardiac sarcoidosis using positron emission tomography/magnetic resonance imaging. Eur Heart J.

[104] Laurent C, Ricard L, Fain O, Buvat I, Adedjouma A, Soussan M (2019). PET/MRI in large-vessel vasculitis: clinical value for diagnosis and assessment of disease activity. Sci Rep.

[105] Einspieler I, Thurmel K, Pyka T, Eiber M, Wolfram S, Moog P (2015). Imaging large vessel vasculitis with fully integrated PET/MRI: a pilot study. Eur J Nucl Med Mol Imaging.

[106] Fernandez-Friera L, Fuster V, Lopez-Melgar B, Oliva B, Sanchez-Gonzalez J, Macias A (2019). Vascular inflammation in subclinical atherosclerosis detected by hybrid PET/MRI. J Am Coll Cardiol.

[107] Robson PM, Dweck MR, Trivieri MG, Abgral R, Karakatsanis NA, Contreras J (2017). Coronary artery PET/MR imaging: feasibility, limitations, and solutions. JACC Cardiovasc Imaging.

[108] Li X, Heber D, Leike T, Beitzke D, Lu X, Zhang X (2018). [68Ga]Pentixafor-PET/MRI for the detection of chemokine receptor 4 expression in atherosclerotic plaques. Eur J Nucl Med Mol Imaging.

[109] Bucerius J, Barthel H, Tiepolt S, Werner P, Sluimer JC, Wildberger JE (2017). Feasibility of in vivo (18)F-florbetaben PET/MR imaging of human carotid amyloid-beta. Eur J Nucl Med Mol Imaging.

[110] Trivieri MG, Dweck MR, Abgral R, Robson PM, Karakatsanis NA, Lala A (2016). (18)F-sodium fluoride PET/MR for the assessment of cardiac amyloidosis. J Am Coll Cardiol.

[111] Erba PA, Habib G, Glaudemans WJM, Miro JM, Slart RHJ (2017). The round table approach in infective endocarditis & cardiovascular implantable electronic devices infections: make your e-team come true. Eur J Nucl Med Mol Imaging.

[112] European Nuclear Medicine Guide. 2018. Available from: https://www.eanm.org/publicpress/european-nuclear-medicine-guide/.

[113] Emsen B, Benali K, Mahida B, Lariviere D, Le Guludec D, Papo T (2018). Comparison between visual and numerical metrics for the evaluation of patients with Takayasu arteritis with 18F-FDG-PET. Nucl Med Commun.

[114] Grayson PC, Alehashemi S, Bagheri AA, Civelek AC, Cupps TR, Kaplan MJ (2018). (18) F-fluorodeoxyglucose-positron emission tomography as an imaging biomarker in a prospective, longitudinal cohort of patients with large vessel Vasculitis. Arthritis Rheumatol.

[115] Han Q, Liang Q, Kang F, Wang J, Wu Z, Zhu P (2018). An increased major vessel uptake by 18F-FDG-PET/CT in NIH criteria inactive patients with Takayasu’s arteritis. Clin Exp Rheumatol.

[116] Imfeld S, Rottenburger C, Schegk E, Aschwanden M, Juengling F, Staub D (2018). [18F]FDG positron emission tomography in patients presenting with suspicion of giant cell arteritis-lessons from a vasculitis clinic. Eur Heart J Cardiovasc Imaging.

[117] Soriano A, Pazzola G, Boiardi L, Casali M, Muratore F, Pipitone N (2018). Distribution patterns of 18F-fluorodeoxyglucose in large vessels of Takayasu’s and giant cell arteritis using positron emission tomography. Clin Exp Rheumatol.

[118] Vaidyanathan S, Chattopadhyay A, Mackie SL, Scarsbrook AF (2018). Comparative effectiveness of (18)F-FDG PET-CT and contrast-enhanced CT in the diagnosis of suspected large-vessel vasculitis. Br J Radiol.

[119] Hohmann C, Michels G, Schmidt M, Pfister R, Mader N, Ohler M (2019). Diagnostic challenges in infective endocarditis: is PET/CT the solution?. Infection..

[120] Graziosi M, Nanni C, Lorenzini M, Diemberger I, Bonfiglioli R, Pasquale F (2014). Role of (1)(8)F-FDG PET/CT in the diagnosis of infective endocarditis in patients with an implanted cardiac device: a prospective study. Eur J Nucl Med Mol Imaging.

[121] Diemberger I, Bonfiglioli R, Martignani C, Graziosi M, Biffi M, Lorenzetti S (2019). Contribution of PET imaging to mortality risk stratification in candidates to lead extraction for pacemaker or defibrillator infection: a prospective single center study. Eur J Nucl Med Mol Imaging.

[122] Granados U, Fuster D, Pericas JM, Llopis JL, Ninot S, Quintana E (2016). Diagnostic accuracy of 18F-FDG PET/CT in infective endocarditis and implantable cardiac electronic device infection: a cross-sectional study. J Nucl Med.

[123] Kumita S, Yoshinaga K, Miyagawa M, Momose M, Kiso K, Kasai T (2019). Recommendations for (18)F-fluorodeoxyglucose positron emission tomography imaging for diagnosis of cardiac sarcoidosis-2018 update: Japanese Society of Nuclear Cardiology recommendations. J Nucl Cardiol.

[124] Tuominen H, Haarala A, Tikkakoski A, Korkola P, Kahonen M, Nikus K (2019). (18)F-FDG-PET in Finnish patients with clinical suspicion of cardiac sarcoidosis: female sex and history of atrioventricular block increase the prevalence of positive PET findings. J Nucl Cardiol.

[125] Tuominen H, Haarala A, Tikkakoski A, Kahonen M, Nikus K, Sipila K (2020). 18-FDG-PET in a patient cohort suspected for cardiac sarcoidosis: right ventricular uptake is associated with pathological uptake in mediastinal lymph nodes. J Nucl Cardiol.

[126] Lee JH, Lee GY, Kim SJ, Kim KH, Jeon ES, Lee KH (2015). Imaging findings and literature review of (18)F-FDG PET/CT in primary systemic AL amyloidosis. Nucl Med Mol Imaging.

[127] Oliveira-Santos M, Castelo-Branco M, Silva R, Gomes A, Chichorro N, Abrunhosa A (2017). Atherosclerotic plaque metabolism in high cardiovascular risk subjects - a subclinical atherosclerosis imaging study with (18)F-NaF PET-CT. Atherosclerosis..

[128] Dweck MR, Chow MW, Joshi NV, Williams MC, Jones C, Fletcher AM (2012). Coronary arterial 18F-sodium fluoride uptake: a novel marker of plaque biology. J Am Coll Cardiol.

[129] Ishiwata Y, Kaneta T, Nawata S, Hino-Shishikura A, Yoshida K, Inoue T (2017). Quantification of temporal changes in calcium score in active atherosclerotic plaque in major vessels by (18)F-sodium fluoride PET/CT. Eur J Nucl Med Mol Imaging.

[130] Quirce R, Martinez-Rodriguez I, Banzo I, Jimenez-Bonilla J, Martinez-Amador N, Ibanez-Bravo S (2016). New insight of functional molecular imaging into the atheroma biology: 18F-NaF and 18F-FDG in symptomatic and asymptomatic carotid plaques after recent CVA. Preliminary results. Clin Physiol Funct Imaging.

[131] Blomberg BA, Thomassen A, de Jong PA, Simonsen JA, Lam MG, Nielsen AL (2015). Impact of personal characteristics and technical factors on quantification of sodium 18F-fluoride uptake in human arteries: prospective evaluation of healthy subjects. J Nucl Med.

[132] Martineau P, Finnerty V, Giraldeau G, Authier S, Harel F, Pelletier-Galarneau M. Examining the sensitivity of 18F-NaF PET for the imaging of cardiac amyloidosis. J Nucl Cardiol. 2019. 10.1007/s12350-019-01675-2 30834499

[133] Voo S, Kwee RM, Sluimer JC, Schreuder FH, Wierts R, Bauwens M, et al. Imaging intraplaque inflammation in carotid atherosclerosis with 18F-fluorocholine positron emission tomography-computed tomography: prospective study on vulnerable atheroma with immunohistochemical validation. Circ Cardiovasc Imaging. 2016;9(5):10.1161/CIRCIMAGING.115.004467. 10.1161/CIRCIMAGING.115.004467 27162131

[134] Verweij N, Bruijnen S, Gent Y, Huisman M, Jansen G, Molthoff C, et al. [18F]FLUORO-PEG-FOLATE pet: a novel imaging technique to visualize rheumatoid arthritis. Annals of the Rheumatic Disease. 2017.

[135] Norikane T, Yamamoto Y, Maeda Y, Noma T, Dobashi H, Nishiyama Y. Comparative evaluation of (18)F-FLT and (18)F-FDG for detecting cardiac and extra-cardiac thoracic involvement in patients with newly diagnosed sarcoidosis. EJNMMI Res 2017;7(1):69–0. 10.1186/s13550-017-0321-0 PMC5574834 28853043

[136] Rayamajhi SJ, Mittal BR, Maturu VN, Agarwal R, Bal A, Dey P (2016). (18)F-FDG and (18)F-FLT PET/CT imaging in the characterization of mediastinal lymph nodes. Ann Nucl Med.

[137] Ehman EC, El-Sady MS, Kijewski MF, Khor YM, Jacob S, Ruberg FL (2019). Early detection of multiorgan light-chain amyloidosis by whole-body (18)F-florbetapir PET/CT. J Nucl Med.

[138] Baratto L, Park SY, Hatami N, Gulaka P, Vasanawala S, Yohannan TK (2018). (18)F-florbetaben whole-body PET/MRI for evaluation of systemic amyloid deposition. EJNMMI Res.

[139] Law WP, Wang WY, Moore PT, Mollee PN, Ng AC (2016). Cardiac amyloid imaging with 18F-florbetaben PET: a pilot study. J Nucl Med.

[140] Zelt JGE, Mielniczuk LM, Orlandi C, Robinson S, Hadizad T, Walter O (2019). PET imaging of sympathetic innervation with [(18)F]flurobenguan vs [(11)C]mHED in a patient with ischemic cardiomyopathy. J Nucl Cardiol.

[141] Sinusas AJ, Lazewatsky J, Brunetti J, Heller G, Srivastava A, Liu YH (2014). Biodistribution and radiation dosimetry of LMI1195: first-in-human study of a novel 18F-labeled tracer for imaging myocardial innervation. J Nucl Med.

[142] Pizarro C, Kluenker F, Dabir D, Thomas D, Gaertner FC, Essler M (2018). Cardiovascular magnetic resonance imaging and clinical performance of somatostatin receptor positron emission tomography in cardiac sarcoidosis. ESC Heart Fail.

[143] Rominger A, Saam T, Vogl E, Ubleis C, la Fougere C, Forster S (2010). In vivo imaging of macrophage activity in the coronary arteries using 68Ga-DOTATATE PET/CT: correlation with coronary calcium burden and risk factors. J Nucl Med.

[144] Minamimoto R, Awaya T, Iwama K, Hotta M, Nakajima K, Hirai R (2020). Significance of (11)C-PIB PET/CT in cardiac amyloidosis compared with (99m)Tc-aprotinin scintigraphy: a pilot study. J Nucl Cardiol.

